# Reprogramming of Amino Acid Metabolism in Pancreatic Cancer: Recent Advances and Therapeutic Strategies

**DOI:** 10.3389/fonc.2020.572722

**Published:** 2020-09-29

**Authors:** Ruiyuan Xu, Jinshou Yang, Bo Ren, Huanyu Wang, Gang Yang, Yuan Chen, Lei You, Yupei Zhao

**Affiliations:** Department of General Surgery, Peking Union Medical College, Peking Union Medical College Hospital, Chinese Academy of Medical Sciences, Beijing, China

**Keywords:** pancreatic cancer, amino acid metabolism, tumor microenvironment, metastasis, angiogenesis, redox balance

## Abstract

Pancreatic ductal adenocarcinoma (PDAC) is one of the most fatal malignancies with an extremely poor prognosis. Energy metabolism reprogramming, an emerging hallmark of cancer, has been implicated in the tumorigenesis and development of pancreatic cancer. In addition to well-elaborated enhanced glycolysis, investigating the role of reprogramming of amino acid metabolism has sparked great interests in recent years. The rewiring amino acid metabolism orchestrated by genetic alterations contributes to pancreatic cancer malignant characteristics including cell proliferation, invasion, metastasis, angiogenesis and redox balance. In the unique hypoperfused and nutrient-deficient tumor microenvironment (TME), the interactions between cancer cells and stromal components and salvaging processes including autophagy and macropinocytosis play critical roles in fulfilling the metabolic requirements and supporting growth of PDAC. In this review, we elucidate the recent advances in the amino acid metabolism reprogramming in pancreatic cancer and the mechanisms of amino acid metabolism regulating PDAC progression, which will provide opportunities to develop promising therapeutic strategies.

## Introduction

Pancreatic ductal adenocarcinoma (PDAC), the most common type of pancreatic cancer, is the most lethal malignancy with a 5-year survival rate of <9%. PDAC is the fourth leading cause of cancer-related death in the United States (1). It was estimated that there were 458,918 new cases being diagnosed and 432,242 deaths emerged in patients with pancreatic cancer worldwide in 2018 (2). The low survival rate of PDAC is associated with the lack of early typical symptoms. Most patients only presented anorexia and weight loss at early PDAC stages and are not diagnosed until at advanced stages or with distant metastases, which resulted in the loss of the best operative opportunity. Currently, surgical resection is the only curative manner for PDAC and advanced patients can only receive conservative treatments such as FOLFIRINOX chemotherapy. Correspondingly, numerous therapeutics including chemotherapy, radiotherapy, immunotherapy, and targeted therapy have not shown significant improvement in long-term survival rate of patients with PDAC (3). The high aggressiveness of PDAC is attributed to the ample desmoplastic microenvironment which plays a critical role in tumor growth, invasion, metastasis, angiogenesis, immunosuppression, and chemoresistance. Therefore, a better understanding of the complex biological characteristics of pancreatic cancer is necessary.

Proliferating cancer cells usually exhibit energy metabolism adjustments to support the rapid cell growth and division, which is an emerging hallmark of cancer known as “energy metabolism reprogramming” that is closely related to PDAC malignant biological behaviors (4). Existing studies suggest that aerobic glycolysis and abnormal lipid metabolism play important roles in pancreatic cancer progression (5, 6). In addition to glucose and lipid metabolism reprogramming, aberrant amino acids metabolism also contributes to fuel fast growth and proliferation of cancer cells. There are 20 standard amino acids to be used for synthesizing proteins in adult body with 8 called essential amino acids (EAAs) and the others called non-essential amino acids (NEAAs). Among the NEAAs, several conditionally essential amino acids such as arginine, cysteine, glycine, glutamine, proline, and tyrosine become dietarily required under special pathophysiological conditions such as cancerous diseases (7). For instance, glutamine, the most abundant amino acid in blood, has been well-investigated to participate in multiple biological processes which are required for cancer cell growth and proliferation. Glutamine can replenish the tricarboxylic acid (TCA) cycle as an anaplerotic substrate and is also the indispensable nitrogen donor for the biosynthesis of purines, pyrimidines, NEAAs, nicotinamide adenine dinucleotide (NAD), and glucosamine. In addition to providing carbon and nitrogen for macromolecular synthesis in cancer cells, glutamine also drives the uptake of EAAs and activates the mammalian target of rapamycin (mTOR) to promote tumor growth (8). Notably, glutamine is essential to maintain redox homeostasis and support tumor growth of PDAC cells in an oncogenic KRAS-driven manner (9). In recent years, increasing studies focus on the amino acids metabolism in PDAC development and progression, which can be mediated by metabolic alterations, redox control, and epigenetic regulation (10–12). More importantly, based on the above energy metabolism reprogramming, pharmacologic and dietary interventions targeting deregulated cancer metabolism has been considered for clinical therapies (13). In this review, we formulate the amino acid metabolism reprogramming in pancreatic cancer and the mechanisms of amino acid metabolism regulating PDAC progression. Finally, we discuss the therapeutic strategies of targeting PDAC amino acid metabolism.

## Regulation of the Reprogrammed Amino Acid Metabolism in PDAC

It has been well-demonstrated that both genetic alterations and unique microenvironment determine the development of pancreatic cancer (14, 15). Whole genome sequencing studies have confirmed the genomic alterations in the progression of early pancreatic intraepithelial neoplasia (PanIN) lesions to metastatic PDAC. *KRAS* mutation is an initiating and driver gene during pancreatic cancer development and is found to mutate the most frequently in over 90% PDAC cases. Moreover, subsequent inactivation of the tumor suppressor genes *CDKN2A, TP53*, and *SMAD4* are also frequently observed during PDAC progression (16, 17). Increasing evidence has demonstrated that pancreatic tumor microenvironment (TME) plays an essential role in PDAC progression and therapeutic resistance. Given the emerging role of the cellular metabolism reprogramming in tumorigenesis and progression, it is logical to conclude that genetic alterations and TME related to PDAC development also participate in the metabolic rewiring process. Therefore, investigating the effects of genetic alterations and interplay between cancer cells and microenvironmental components on amino acid metabolism reprogramming helps to better understand PDAC biological characteristics.

### Genetic Alterations

#### KRAS

In humans, three *RAS* genes including *HRAS, NRAS*, and *KRAS* encode four highly homologous ~21 kDa small GTPases: HRAS, NRAS, KRAS4A, and KRAS4B. Activated RAS proteins contribute to many of malignant hallmarks of tumor including promotion of proliferation, suppression of apoptosis, metabolism reprogramming, remodeling the microenvironment, evasion of the immune response, and acquisition of metastatic properties (18). Among the different *RAS* isoforms, *KRAS* mutation is found in over 90% PDAC. Proliferating cancer cells require increased uptake of glutamine for their excessive need, making it conditionally essential for the growth of many types of cancer. Glutamine-derived glutamate supports the viability and proliferation of cancer cells by replenishing tricarboxylic acid (TCA) cycle intermediate α-ketoglutarate (α-KG) that is mediated by either glutamate dehydrogenase (GLUD) or aminotransferases alanine aminotransferase (also known as glutamate-pyruvate transaminase, GPT) and aspartate aminotransferase (also known as glutamate-oxaloacetate transaminase, GOT) (8). Son et al. identified a non-canonical glutamine metabolism pathway in a KRAS-driven GOT1-malate dehydrogenase 1 (MDH1)-malic enzyme 1 (ME1)-mediated manner in PDAC, which is critical to maintain redox homeostasis. Notably, the expression of GOT1 increased and GLUD1 decreased in an inducible oncogenic KRAS PDAC mouse model, further supporting the notion that KRAS plays a key role in shifting glutamine metabolic pathways in PDAC (9). A recent study suggests that methylation on arginine 248 inhibits MDH1 catalytic activity and dimerization by coactivator-associated arginine methyltransferase 1 (CARM1), and KRAS suppresses CARM1-mediated MDH1 methylation, contributing to glutamine metabolism in pancreatic cancer (19). Additionally, oncogenic KRAS-induced NRF2 could upregulate glutaminolysis through increasing the expression of major glutamine metabolism intermediates such as GLS1, GOT1, and Na^+^-independent cystine/glutamate antiporter SLC7A11 (also known as xCT) (20).

**Figure 1:**
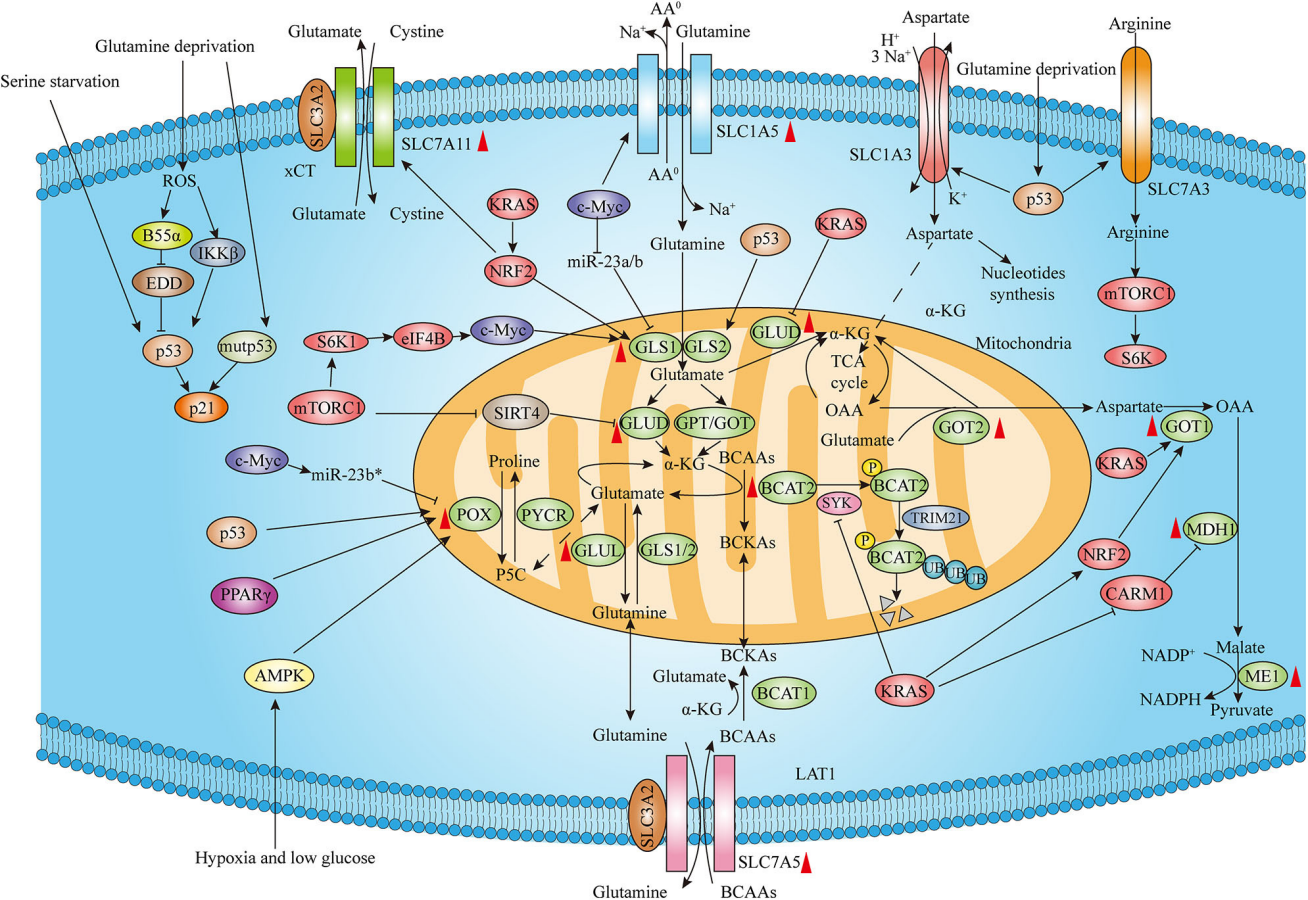
Genetic alterations regulate amino acid metabolism reprogramming in PDAC. The expression of amino acid metabolism intermediates including rate-limiting enzymes and transporters is associated with distinct genetic alterations such as KRAS, MYC, p53 as well as other genetic mutations and signaling regulators. Moreover, both wild-type p53 and mutant p53 contribute to the cellular adaptions to amino acids deprivation. Red upward arrowheads next to the amino acids metabolism enzymes and transporters represent upregulating expression in pancreatic cancer tissues (The expression of GLUD is atypical and AA^0^ means neutral amino acids).

The branched-chain amino acids (BCAAs) leucine, isoleucine, and valine are EAAs. BCAAs can be transported by a Na^+^-independent systemic L amino acid transporter SLC7A5 (also known as LAT1). BCAA catabolism is mediated by the cytosolic branched-chain amino acid transaminase 1 (BCAT1) and mitochondrial branched-chain amino acid transaminase 2 (BCAT2) which transfer the amino groups from BCAAs to α-KG to produce branched-chain α-keto acids (BCKAs) and glutamate. BCAA breakdown can not only provide carbon for synthesis of metabolites to fuel TCA cycle which can contribute to energy production but also supply nitrogen for *de novo* nucleotide and non-essential amino acid biosynthesis in cancer (21). It has been shown that plasma BCAAs levels are elevated in early-stage pancreatic cancers driven by mutant KRAS (22). A recent study demonstrated that BCAT2, but not BCAT1, was overexpressed in PanIN and PDAC ductal cells. The authors also investigated the effect of KRAS mutation on the expression of BCAT2 and found that KRAS stabilizes BCAT2 by inhibiting spleen tyrosine kinase (SYK) induced tyrosine 228 phosphorylation and subsequent tripartite-motif-containing protein 21 (TRIM21) E3 ligase-mediated BCAT2 degradation. Thus, the study highlights that BCAA-BCAT2 axis driven by KRAS is critical for development of PDAC (23) (Figure 1).

#### MYC

The MYC oncogene, which is frequently deregulated among multiple human malignancies, encodes the oncogenic transcription factor c-Myc to drive tumorigenesis associated with cellular proliferation, DNA replication and transcription, protein synthesis and altered tumor cell metabolism (24, 25). C-Myc is overexpressed in many PDAC cases and exerts as a master regulator of essential cellular processes (26). Recently, emerging studies have provided evidence on effects of c-Myc regulating amino acids especially glutamine metabolism in PDAC. c-Myc has been shown to increase levels of Na^+^-dependent glutamine transporter SLC1A5 (also known as ASCT2) by binding to its promoter region, leading to elevated uptake of glutamine (27). Moreover, c-Myc also regulates glutamine catabolism through increasing mitochondrial glutaminase (GLS) expression which converts glutamine to glutamate. The mechanism of c-Myc enhancing GLS is through c-Myc-mediated suppression of microRNAs miR-23a and miR-23b (28). In addition to modulating glutamine metabolism directly, other signaling pathways are capable of regulating c-Myc activity. The mammalian target of rapamycin complex 1 (mTORC1)/S6K1 pathway has been indicated to positively regulate GLS and glutamine flux through the eIF4B-dependent regulation of c-Myc (29). Furthermore, Deng et al. recently reported a novel long non-coding RNA (lncRNA)-mediated reciprocal feedback loop of Myc and GLS in pancreatic cancer. They suggested that an antisense lncRNA of glutaminase (GLS-AS) could be transcriptionally inhibited by Myc, leading to GLS upregulation during the deprivation of glucose and glutamine. In turn, GLS-AS decreased Myc expression via impairment of the GLS-mediated stabilization of Myc (30). Recently, the other lncRNA XLOC_006390 was demonstrated to increase GLUD1 expression by binding to and stabilizing c-Myc, enhancing α-KG production to replenish TCA cycle and promote PDAC progression (31).

Proline, the other NEAA, has also been indicated to play important roles in metabolism reprogramming of cancer. Phang's group has emphasized the metabolic link between glutamine and proline controlled by c-MYC in human cancers. Glutamate can be converted to proline through Δ^1^-pyrroline-5-carboxylate (P5C) catalyzed by P5C synthase (P5CS) and subsequent P5C reductase (PYCR). Conversely, proline catabolism mediates the conversion of proline to glutamine through proline oxidase (POX) also known as proline dehydrogenase (PRODH) and Δ^1^-pyrroline-5-carboxylate dehydrogenase (P5CDH) sequentially. It was shown that c-MYC could not only inhibit POX/PRODH expression primarily through increasing miR-23b^*^ but also evidently increase the biosynthesis of proline from glutamine, maintaining cancer cell survival and proliferation (32) (Figure 1).

#### p53

The transcription factor p53 exerts its tumor suppression functions by both inducing genes involved in cell cycle arrest, DNA repair, or apoptosis and regulating other cellular processes such as cell metabolism (33). The role of p53 in glucose metabolism has been well-indicated (34). Moreover, p53 could increase the GLS2 expression to facilitate glutamine metabolism and regulate antioxidant defense function by increasing intracellular reduced glutathione (GSH) levels and decreasing reactive oxygen species (ROS) levels, which protects cells from oxidative stress (35, 36).

However, since poor vascularization in the PDAC microenvironment and increased glutamine catabolism as tumors grow rapidly, tumor cells are frequently exposed to a low glutamine microenvironment. The tumor suppressor wild-type p53 can not only inhibit proliferation but also help cells survive and repair DNA damage (37). Emerging evidence has shown that p53 exerts a critical role in the aberrant metabolism in cancer and can contribute to the cellular adaptions to metabolic stress. Kong's group has reported that cancer cells are able to survive under glutamine deprivation conditions through the activation of p53 and related signaling pathway. The researchers identified that both the protein phosphatase 2A (PP2A) B subunit B55α-E3 ubiquitin ligase identified by differential display (EDD)-p53 pathway and I-kappa-B-kinase β (IKKβ)-p53 signaling axis are essential for cancer cell survival and tumor growth in response to glutamine deprivation (38, 39). Recently, the role of p53 upregulating amino acid transporters in response to glutamine removal has been proven. Kong et al. demonstrated that SLC7A3, an arginine transporter, is induced in a p53-dependent manner following glutamine deprivation, leading to increased intracellular arginine levels. The influx of arginine further contributes to mTORC1 activation and promotes cell proliferation and tumor growth (40). Tajan et al. discovered that p53 enhances the expression of SLC1A3, an Na^+^/K^+^/H^+^-dependent aspartate/glutamate transporter that allows the aspartate metabolism to sustain cancer cell survival and tumor growth under glutamine starvation (41). Additionally, p53 protein is mutated in over 50% of PDAC, and tumor-associated mutant p53 (mutp53) protein has been well-known to drive aggressive cancer growth, invasion, metastasis, and chemotherapy resistance (42). In addition to wild type p53, the role of mutp53 protecting cancer cells from metabolic stress has also been well-established. Kong's group also demonstrated that cancer cells expressing mutp53 proteins are more resistant to low glutamine conditions than cells with wild type p53. Specifically, mutp53 hyper-induces p53 target gene CDKN1A (p21) expression to trigger G1/S cell cycle arrest to promote cell survival from glutamine withdrawal (43). Interestingly, p53 also contributes to cell survival under other amino acid depletion conditions. Maddocks et al. established that p53-induced p21 activation results in cell cycle arrest and enhanced GSH flux, allowing cancer cells to combat oxidative stress and promoting cell survival and proliferation in response to serine depletion (44) (Figure 1).

#### Other Genetic Alterations

SIRT4, a mitochondria-localized sirtuin, has been well-known to inhibit glutamine metabolism and insulin secretion from the pancreatic β cells by inhibiting GLUD (45). Csibi et al. found that mTORC1 represses SIRT4 expression by promoting the proteasome-mediated degradation of cAMP-responsive element binding 2 (CREB2), resulting in promoting glutamine anaplerosis by activating GLUD (46). Moreover, Haigis and colleagues reported that SIRT4 functions as a tumor suppressor to regulate the cellular metabolic response to DNA damage by suppressing mitochondrial glutamine metabolism (47). Indeed, a variety of human cancers including lung, bladder, gastric, and breast cancers as well as leukemia have decreased SIRT4 expression and lower SIRT4 level was associated with poorer prognosis (46, 47). Recently, SIRT4 has been demonstrated to inhibit PDAC cell proliferation and serves as a negative regulator of aerobic glycolysis in pancreatic cancer (48). Hence, whether SIRT4 exhibits tumor suppressive functions to negatively regulate glutamine metabolism by inhibiting GLUD in pancreatic cancer requires more research. Tumor suppressor gene *SMAD4* is homozygously deleted in nearly one-third of PDAC and deletion of *SMAD4* can be associated with the loss of its neighboring housekeeping gene *malic enzyme 2 (ME2)*. Dey et al. reveal that genomic deletion of *ME2* confers collateral lethality in pancreatic cancer via regulation of BCAA metabolism. In *ME2*-null PDAC cells, *ME3* depletion leads to ROS accumulation and activation of the AMP-activated protein kinase (AMPK), which suppresses sterol regulatory element-binding protein 1 (SREBP1)-directed transcription of BCAT2, thereby resulting in a decrease in *de novo* nucleotide biosynthesis (49). In addition, Mayers et al. demonstrated that the same genetic event can result in distinct BCAA metabolism by establishing the mouse models of PDAC and non-small cell lung carcinoma (NSCLC) both driven by KRAS mutation and Trp53 deletion. In contrast to mice with early PDAC, mice with early NSCLC exhibited decreased plasma BCAAs levels. NSCLC tumors actively took up and catabolized BCAAs to provide nitrogen for non-essential amino acids and nucleotide synthesis, whereas expression of BCAA catabolism pathway enzymes was decreased in PDAC tumors (50). Collectively, above studies indicate that both genetic mutation and tissue-of-origin can influence BCAA metabolism in PDAC.

Moreover, existing studies revealed that same amino acid metabolism enzyme with distinct signaling regulators displays diverse effects in cancer cells. Phang's group conducting a series of experiments proves that proline metabolized by POX to generate ROS critically contributes to inducing apoptosis as well as autophagy. In colon cancer cells, both peroxisome proliferator-activated receptor gamma (PPARγ) activation and p53 induction upregulate expression of POX, leading to the ROS formation and cell apoptosis (51). Furthermore, deprivation of both oxygen and glucose can induce the AMPK activation and subsequent POX upregulation, which results in ROS and ATP production under hypoxic and low-glucose conditions, respectively. Both POX-induced ROS and ATP eventually promote colon cancer cell survival through ROS-induced protective autophagy and direct energy supply (52). Collectively, above studies indicate that distinct effects of proline metabolism by POX depend on the existing metabolic conditions as well as upstream signaling pathway and the effect of POX mediating proline metabolism in PDAC deserves further exploration (Figure 1).

### Tumor Microenvironment (TME)

The pancreatic TME consists of cancer cells, stromal cells, and extracellular components. Pancreatic cancer is characterized by dense desmoplasia, resulting in a considerable nutrient-limiting and hypoxic environment. Despite the effect of hypoxia in promoting glycolysis has been well-verified (53–55), the effect of hypoxia and hypoxia-induced factor (HIF) on glutamine metabolism is still unknown in PDAC. A recent study has indicated that PI3K/mTORC2 pathway increases GOT1 expression and stimulates non-canonical glutamine metabolism by targeting HIF-2α, promoting the progression of PDAC both *in vitro* and *in vivo* (56). Moreover, Yoo et al. recently identified that the SLC1A5 variant is a mitochondrial glutamine transporter which is induced by hypoxia activating HIF-2α. Notably, the SLC1A5 variant acts an oncogenic role in mediating glutamine-induced ATP production, regulating cellular redox homeostasis and conferring gemcitabine resistance to pancreatic cancer cells, therefore promoting PDAC growth (57).

**Figure 2:**
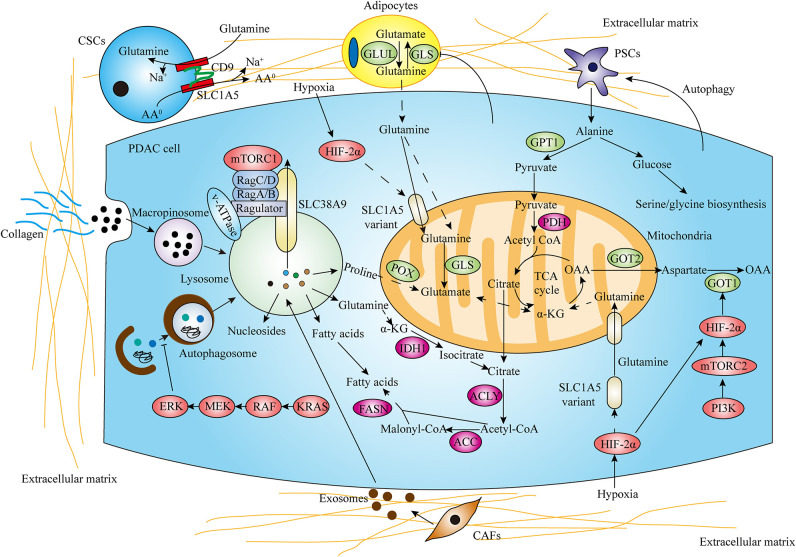
Tumor microenvironment (TME) and salvaging processes promote amino acid metabolism reprogramming in PDAC. In the hypoxic and nutrient-limiting pancreatic microenvironment, the interactions between cancer cells and stromal components including pancreatic stellate cells (PSCs), cancer-associated fibroblasts (CAFs), cancer stem cells (CSCs), adipocytes and collagen strikingly influence PDAC amino acid metabolism. Autophagy and macropinocytosis also play critical roles in recycling and scavenging nutrients to fuel metabolic requirements (AA^0^ means neutral amino acids).

Furthermore, the interactions between cancer cells and stromal components also critically contribute to metabolic reprogramming in PDAC. As the most prominent component in TME, pancreatic stellate cells (PSCs) strikingly influence PDAC metabolism through forming the metabolic crosstalk with cancer cells, thereby promoting tumor cell proliferation and invasion under nutrient-deprived conditions (58, 59). A recent study has demonstrated that PDAC cells stimulate autophagy in PSCs and mediate PSCs secreting alanine. PSCs-derived alanine exerts functions in acting as an alternative carbon source to glucose and glutamine to fuel TCA cycle, support lipid and NEAAs biosynthesis and shunts glucose to serine/glycine biosynthesis, promoting PDAC cells growth in nutrient-limited conditions (11). Cancer-associated fibroblasts (CAFs), which develops mostly from activated PSCs, has been shown to release exosomes to promote proliferation and drug resistance of PDAC (60). Moreover, exosomes derived from CAFs supply metabolites (including TCA cycle metabolites, amino acids, and lipids) in a KRAS independent manner and increase the reductive glutamine metabolism, enhancing PDAC cells proliferation (61). The role of other stromal cell types focusing on glutamine metabolism has also been indicated. Cancer stem cells (CSCs) are characterized with enhanced proliferative capacity, self-renewal ability, metastatic potential, therapy resistance, and generating cellular heterogeneity (62). Recently, it was demonstrated that CD9 identifies CSCs that increase tumor formation capability and recapitulate the cellular heterogeneity of primary PDAC. Mechanistically, CD9 expression enhances glutamine uptake by interacting with and increasing the expression of ASCT2, thereby promoting PDAC growth (63). Peri-tumor adipocytes are correlated with poor outcomes in PDAC (64). The role of adipocytes supplying PDAC cells with glutamine in nutrient-limited PDAC microenvironment has been indicated. Adipocyte-induced PDAC cell proliferation is through a mechanism by which PDAC cells decrease GLS expression in adipocytes and increase glutamine secretion (65). Given that hypoxic and nutrient-limited environment of PDAC is characterized by a tight desmoplasia with a dense collagen meshwork and proline constitutes the predominant components in collagen, a recent study demonstrated that PDAC cells use collagen-derived proline to promote cell survival and proliferation via TCA cycle metabolism under nutrient limited conditions and POX-mediated proline metabolism promotes pancreatic tumor growth (66). Given the critical roles of metabolic crosstalk between cancer cells and stromal components, targeting TME may be a potential therapeutic approach for PDAC treatment (Figure 2).

### Autophagy and Macropinocytosis

Pancreatic cancer cellular metabolism adaption is required for cancer cell survival in a harsh environment where oxygen and nutrients are scarce. Meanwhile, autophagy and macropinocytosis play critical roles for recycling and scavenging nutrients in pancreatic cancer. Autophagy is a regulated catabolic process through lysosomal degradation of intracellular organelles and macromolecules to maintain metabolic and cellular homeostasis. Pancreatic cancer displays elevated autophagy under basal conditions and inhibition of autophagy by genetic or pharmacologic means leads to elevated DNA damage, increased ROS, and decreased mitochondrial oxidative phosphorylation, resulting in significant growth inhibition of PDAC *in vitro* and *in vivo* (67). In PDAC cells, increased MiT/TFE proteins nuclear import drives the autophagy-lysosome genes expression and MiT/TFE-dependent autophagy-lysosome activation is required to maintain intracellular amino acid pools and PDAC growth (68). Interestingly, a recent study indicated that suppression of KRAS or its effector ERK MAPK increased autophagic flux in part by impairing KRAS- or ERK-driven glycolytic and mitochondrial functions in PDAC (69). In addition to utilizing autophagy to recycle cellular metabolites, PDAC cells have the potential to take up and internalize extracellular fluid to fuel elevated metabolic demand. Macropinocytosis is a conserved endocytic process that results in non-specific bulk internalization of extracellular macromolecules into the cell through macropinosomes. In KRAS expressing PDAC cells, transporting extracellular protein via macropinocytosis degrades in the lysosome to produce amino acids, which contributes to the central carbon metabolism (70). Recent studies also demonstrated that macropinocytosis contributes to the supply of free amino acid levels within pancreatic tumors *in vivo* (71, 72).

mTORC1, a well-known growth regulator, is commonly activated in tumors and drives the metabolic reprogramming of cancer cells to support biosynthetic needs for rapid proliferation (73). A recent study revealed that a lysosomal transporter SLC38A9 as an essential part of the Ragulator-RAG GTPases shows high glutamine transport activity to stimulate mTORC1 (74). In pancreatic cancer, SLC38A9 mediates the lysosomal efflux of many EAAs including leucine in an arginine-regulated fashion and promotes mTORC1 activation, supporting cell proliferation, and tumor growth (75). Accumulating evidence has demonstrated that mTORC1 senses diverse environmental conditions including amino acids. When free amino acids are sufficient in the extracellular microenvironment, their uptake through transporters results in activation of mTORC1. mTORC1 activation leads to autophagy inhibition and suppression of degradation of extracellular proteins via macropinocytosis. However, in amino acids depleted conditions, mTORC1 inhibition induces continued tumor growth through autophagy and macropinocytosis (76–78). In addition, Nofal et al. further demonstrated that amino acid scarcity could induce protein scavenging via an mTORC1-independent manner and mTOR inhibition enhances protein-scavenging cell growth in part by limiting translation and restoring amino acid balance in nutrient-deprived conditions (79). Moreover, since both autophagy and macropinocytosis degrade nutrients at the lysosome, blocking lysosomal acidification drugs including chloroquine and its derivative hydroxychloroquine may be effective treatments for PDAC (Figure 2).

## Amino Acid Metabolism Regulates PDAC Development and Progression

**Figure 3:**
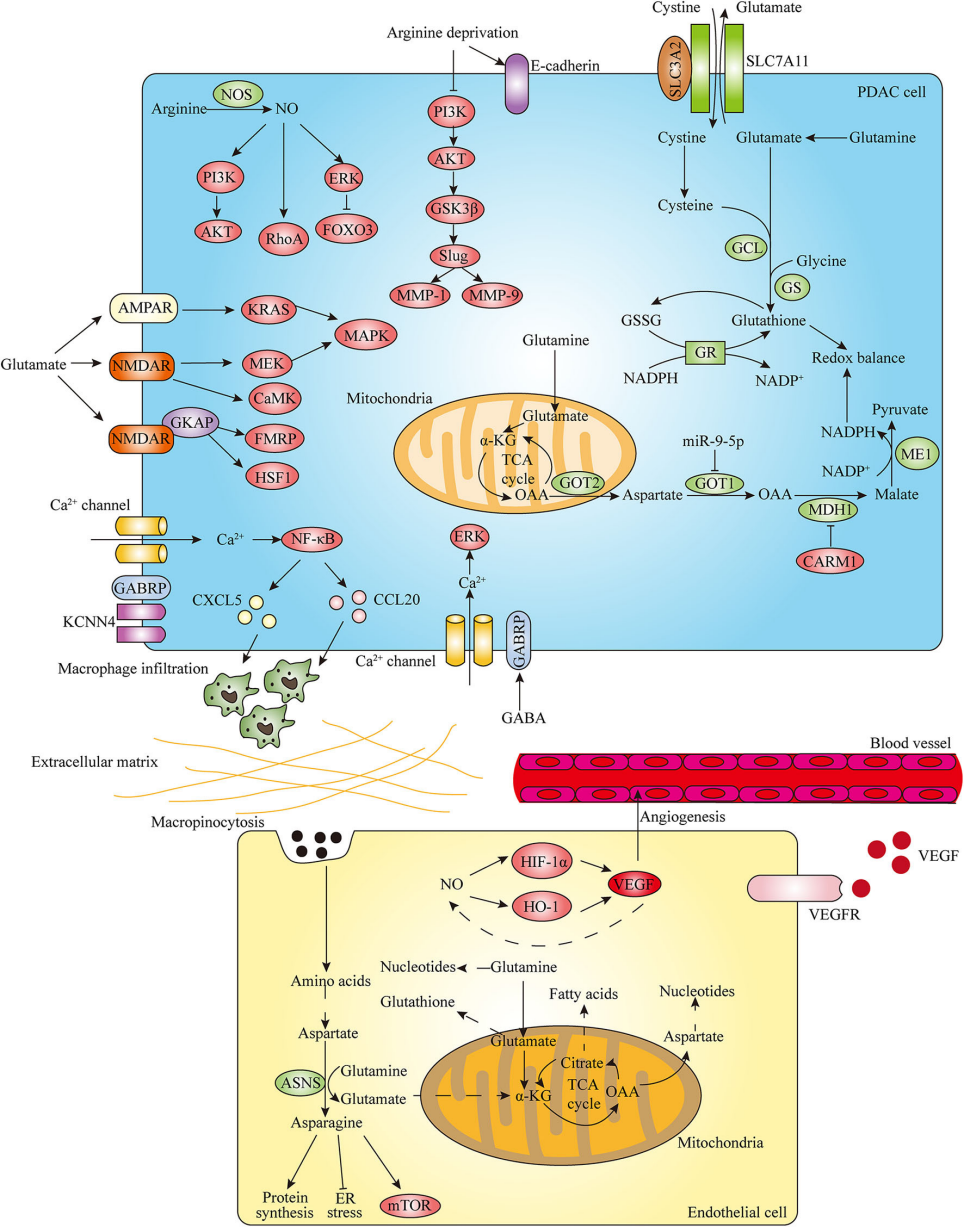
Amino acid metabolism regulates PDAC progression. In the development of PDAC, amino acid metabolism plays important roles in promoting invasion and metastasis, stimulating angiogenesis and regulating redox balance. Arginine metabolism contributes to invasion and migration of pancreatic cancer cells through modulating EMT-inducing transcription factors (EMT-TFs) expression and nitric oxide (NO)-mediated signaling pathways. In addition, glutamate and GABA related specific receptors can also regulate PDAC invasive properties through activating downstream effectors. In endothelial cells (ECs), glutamine metabolism is essential to support cell proliferation and sprouting by interlinking with asparagine metabolism. The cooperative interplay between NO and VEGF also contributes to angiogenesis. In PDAC, glutamine plays critical roles in regulating cellular redox balance by generating glutathione and NADPH. Distinct signaling factors can have impacts on pancreatic cancer cell redox balance by regulating the glutamine metabolic pathway.

Emerging evidence has shown that amino acid metabolism plays a vital role in the initiation and progression of pancreatic cancer. Genetic alterations are closely associated with pancreatic cancer tumorigenesis. KRAS mutation, the initiating event involved in PDAC tumorigenesis, is found in low grade PanIN lesions and can induce intraductal papillary mucinous neoplasm (IPMN) formation with inactivation of tumor suppressor genes such as LKB1 and PTEN synergistically (80–82). In addition to KRAS, other genetic alterations related to amino acid metabolism enzymes have been implicated in the pathogenesis of PDAC. Genetic ablation of *Bcat2*, endothelial NOS (*eNOS*), and glutamate ammonia ligase (*GLUL*) could attenuate PanIN progression (23, 83, 84). Moreover, genetic alterations have also been demonstrated to drive tumorigenesis through coupled metabolic and epigenetic reprogramming. Oncogenic KRAS cooperates with LKB1 loss to induce the serine-glycine-one carbon pathway that supports S-adenosyl methionine (SAM) generation and increase the activity of DNA methyltransferase (DNMT), which enhances DNA methylation and promotes pancreatic tumorigenesis (12). Furthermore, pancreatic tumor cells have an increasing demand for diverse amino acids as bioenergetics and biosynthesis substrates to support rapid growth and proliferation (Table 1). In addition to driving tumorigenesis and sustaining proliferative ability, amino acid metabolism is also involved in other processes including regulating invasion, metastasis, angiogenesis, and redox balance which are associated with the development of pancreatic cancer. Here, we summarize the mechanisms by which amino acid metabolism regulates these aspects in PDAC (Figure 3).

**Table 1:** Amino acids regulate pancreatic cancer growth.

**Amino acids**	**Crucial enzymes/transporters**	**Functions**	**References**
Glutamine	GLUL	Promoting cell proliferation and tumor growth	(84)
	GLS	Promoting cell growth and proliferation	(29, 65)
		Facilitating cell proliferation and invasion	(30)
	GOT1	Supporting cell growth	(85)
		Supporting cell and tumor growth	(9, 86)
		Promoting cell proliferation and invasion	(56, 87)
	GOT2	Sustaining cell growth and suppressing senescence	(88)
		Promoting cell proliferation and tumor growth	(89)
	MDH1	Supporting cell and tumor growth	(9)
		Promoting cell proliferation	(19)
	ME1	Supporting cell and tumor growth	(9)
	SLC1A5 variant	Supporting cell and tumor growth	(57)
Branched-chain amino acids	BCAT2	Enhancing cell proliferation and migration; Promoting PanIN formation and tumor growth	(23)
	BCAT2 and BCKDHA	Promoting cell proliferation and tumor growth	(90)
Proline	POX	Promoting cell proliferation and tumor growth	(66)
Cystine/cysteine	SLC7A11	Supporting cell growth	(91)
		Supporting cell and tumor growth; averting ferroptosis	(92, 93)
Alanine	GPT1/2	Promoting cell proliferation and tumor growth	(11)
Arginine	ARG2	Supporting tumor growth	(94)
GABA	GABRP	Stimulating cell proliferation	(95)

### Promotion of Invasion and Metastasis

Activating invasion and metastasis contributes to one of the main hallmarks of cancer (4). Epithelial to mesenchymal transition (EMT), which is a crucial feature of PDAC, occurs in the very early stages of tumor development, leading to early dissemination, drug resistance, and poor prognosis (96, 97). The EMT programs are mediated by master EMT-inducing transcription factors (EMT-TFs), including Snail, Slug, Twist, and Zeb1 (98, 99). It has been demonstrated that Zeb1 promotes pancreatic tumor progression from formation of early precursor lesions toward late-stage metastasis in contrast to EMT-TFs Snai1 and Twist1, which suggests that different EMT-TFs have specific and complementary subfunctions in driving pancreatic tumor metastasis (100, 101).

The progression of pancreatic cancer is highly reliant on amino acids, which can affect EMT program through modulating various EMT-TFs expression. A recent study demonstrated that arginine deprivation inhibited the adhesion, invasion and migration of pancreatic cancer cells through decreasing the expression of Snail, Slug, Twist as well as matrix metalloproteinases (MMPs) MMP-1 and MMP-9 and increasing E-cadherin expression, which is mediated by regulation of the PI3K/AKT/GSK3β signaling axis (102). As the only precursor available for the production of nitric oxide (NO) that modulates different cancer-related events, arginine plays an important role in tumor growth and metastasis. Nitric oxide synthase (NOS) family, which includes neuronal NOS (nNOS or NOS1), inducible NOS (iNOS or NOS2) and endothelial NOS (eNOS or NOS3), catalyzes the conversion of arginine into citrulline and produces NO. The increased expression of iNOS and eNOS has been found in PDAC compared with normal tissue (103, 104). In PDAC, a high level of iNOS is associated with proliferation and invasiveness of tumor cells (105). Furthermore, NO is associated with the invasive phenotype of pancreatic cancer (106, 107). The function of NO and related signaling pathways in the regulation of pancreatic cancer development and progression has also been implicated. NO can contribute to enhanced invasive properties of PDAC cells via activation of the PI3K-AKT, RhoA, and ERK-Forkhead box transcription factor O 3 (FOXO3) pathways (105, 108) (Figure 3).

In addition to arginine and its metabolite NO, glutamine metabolism also plays a vital role in invasive property of pancreatic cancer (Figure 3). It has been indicated that proliferation and invasion of pancreatic cancer cells could be inhibited via suppression of glutamine metabolism enzyme GLS or GOT1 (30, 87). In recent years, emerging evidence suggest that neurotransmitters exert regulatory roles in TME to influence various malignant behaviors of cancer cells. As a crucial metabolite, glutamate is not only an important bioenergetic substrate for proliferating normal and cancer cells, but also a key excitatory neurotransmitter in the central nervous system (CNS). Glutamate exerts its action through activating metabotropic glutamate receptors (mGluRs) and ionotropic glutamate receptors (iGluRs) including N-methyl-D-aspartate (NMDA) receptors (NMDARs), α-amino-3-hydroxy-5-methyl-4-isoxazolepropionate (AMPA) receptors (AMPARs), and kainate receptors (KARs) (109). The role of glutamate as the excitatory neurotransmitter outside CNS particularly in pancreatic cancer is poorly understood. Recently, several studies investigated the effect of glutamate regulating invasiveness of pancreatic cancer via stimulation of its receptors. Glutamate-mediated AMPAR activation was found to increase invasion and migration of pancreatic cancer cells by activating KRAS-MAPK signaling pathway (110). Furthermore, Hanahan's group demonstrated that glutamate-NMDAR signaling pathway also contributes to pancreatic tumor invasion. The expression of NMDAR is upregulated in genetically engineered mouse models (GEMMs) of PDAC and pancreatic neuroendocrine tumor (PNET) (111, 112). Interstitial flow induced autologous glutamate secretion and subsequent activation of NMDAR and its downstream CaMK and MEK-MAPK pathways, thereby promoting invasiveness of PNET (111). Moreover, GKAP acts as a signal modulator of activity of the glutamate-NMDAR pathway and NMDAR/GKAP signaling supports invasiveness of both PDAC and PNET cells through activation of downstream effectors FMRP and HSF1 (112). In addition to glutamate, γ-aminobutyric acid (GABA), a non-protein amino acid synthesized by decarboxylation of glutamate by glutamic acid decarboxylase (GAD), is the main inhibitory neurotransmitter that exerts functions through GABA receptors including the ionotropic GABA_A_ and GABA_C_ receptors and the metabotropic GABA_B_ receptor in the CNS (113, 114). The different effects of GABA_A_ receptor and GABA_B_ receptor on pancreatic cancer metastasis have been implicated. The stimulation of the GABA_B_ receptor has been shown to suppress the invasiveness and metastatic potential of PDAC by inhibiting β-adrenergic signaling (115). Moreover, GABRP is the pi subunit of the GABA_A_ receptor and the mechanism of GABRP regulating progression of pancreatic cancer has been discovered. The expression of GABRP is increased during malignant transformation of PDAC and patients with high GABRP expressing PDAC have a poor prognosis (95, 116). GABA can increase Ca^2+^ influx through GABRP and subsequently activate MAPK/ERK cascade, resulting in the growth promotion of PDAC cells (95). Furthermore, GABRP can also regulate PDAC progression in a GABA-independent manner. It was revealed that GABRP-KCNN4 complex induces a specific Ca^2+^-dependent activation of nuclear factor κB (NF-κB) signaling and further facilitates macrophage infiltration by inducing CXCL5 and CCL20 expression, thereby promoting tumor growth and metastasis in PDAC (116). Hence, the above findings suggest that targeting amino acids-related neurotransmitter receptor signaling pathways can be promising molecular targets for the treatment of pancreatic cancer.

### Stimulation of Angiogenesis

Angiogenesis, the process by which novel blood vessels grow from pre-existing ones, is crucial for growth and metastasis of many tumors including pancreatic cancer by supplying nutrients and oxygen (117). Endothelial cells (ECs) play an essential role in promoting angiogenesis. It has been recognized that new vessel formation induced by ECs is not only dependent on growth factors-induced signaling cascades but also on endothelial metabolic phenotypes (118). During angiogenesis, ECs need to increase their metabolic activity to meet the biomass and bioenergetic demands of cell proliferation and migration. A large body of evidence has demonstrated that ECs are highly glycolytic, and they can take up glucose and produce large amounts of lactate through aerobic glycolysis (119). However, the role of amino acids in endothelial metabolism is unclear. Recent published studies provide evidence into how ECs use amino acids to support cell proliferation and angiogenesis (Figure 3). In ECs, glutamine metabolism via GLS is essential to replenish the TCA cycle, maintain redox balance and produce amino acids, proteins, nucleotides and lipids required for cell proliferation. Moreover, inhibiting asparagine synthetase (ASNS) expression impaired EC sprouting in the presence of glutamine and ECs also use macropinocytosis to provide non-essential amino acids including asparagine under glutamine limitation, which indicates that glutamine metabolism interlinks with asparagine metabolism in vessel sprouting (120, 121). Furthermore, in glutamine-deprived ECs, asparagine not only contributes to rescuing the proliferation defects but also proves crucial in restoring protein synthesis, suppressing endoplasmic reticulum (ER) stress and reactivating mTOR signaling (120). Given that mounting studies have suggested that GLS1 inhibition or blocking ASNS in combination with asparaginase treatment is effective in attenuating tumor growth, targeting GLS1 and ASNS could be promising therapeutic strategies to suppress cancer progression through impairing angiogenesis.

In addition to glutamine and asparagine, arginine metabolite NO and key enzyme NOS have been indicated to regulate angiogenesis and thus exert a significant impact on tumor progression (122). A large body of studies have revealed the role of the interactions between NO and angiogenic factor vascular endothelial growth factor (VEGF) on angiogenesis. NO could upregulate VEGF by activating the transcription factor HIF-1α, thereby promoting angiogenesis (123). Besides HIF-1α, heme oxygenase-1 (HO-1) also participates in NO-induced VEGF production in human umbilical vein endothelial cells (HUVECs) (124). In turn, numerous studies also demonstrated that eNOS contributes to the VEGF-induced angiogenesis via production of NO in ECs (125, 126). The cooperative interplay between NO and VEGF on tumor angiogenesis has been well-proved in various human cancers (127). Moreover, existing studies have also investigated the role of NO implicated in angiogenesis of pancreatic cancer. It has been suggested that increased eNOS expression in the vasculature and peritumoral tissue of PDAC is involved in the vascularization and neovascularization of pancreatic tumors (128). Additionally, the combination of NOS inhibition and VEGF receptor 2 (VEGFR-2) blockade significantly increased the anti-vascular effect over either therapy alone, resulting in greater pancreatic tumor growth inhibition (129). Therefore, based on the above findings, a better understanding of the mechanisms of various amino acids metabolism regulating ECs metabolic phenotypes and tumor angiogenesis will help to develop more effective anti-angiogenic therapy for treating pancreatic cancer.

### Regulation of Redox Balance

Cancer cells encounter high levels of oxidative stress due to accumulated ROS during rapid progression, which enables them to exhibit elevated antioxidant capacity (130). Glutamine plays a critical role in maintaining redox balance of tumor cells (Figure 3). It is suggested that disruption of glutamine metabolism leads to a downregulation of various redox homeostasis proteins and an increase in accumulation of ROS, resulting in cellular redox imbalance to facilitate pancreatic cancer cell apoptosis (131). Glutathione (GSH), a tripeptide comprised of glutamate, cysteine, and glycine, is a key antioxidant molecule which can promote cancer cell redox homeostasis. The synthesis of GSH involves two ATP-dependent steps: formation of γ-glutamylcysteine from glutamine-derived glutamate and cysteine and following formation of GSH from γ-glutamylcysteine and glycine (132). Glutamine-derived glutamate also contributes to GSH synthesis by facilitating the uptake of cystine through the SLC7A11 (also known as xCT) transporter, which is coupled to the efflux of glutamate (133). Subsequently, cystine is converted to cysteine for incorporation into GSH in the cell. It has been revealed that glutamine deprivation could lead to decreased cystine uptake through SLC7A11 and reduced intracellular GSH levels (134, 135). Moreover, the expression of SLC7A11 is upregulated in the pancreatic tumor tissues and pancreatic cancer cells can increase SLC7A11 expression in response to oxidative stress, which results in the increase in GSH synthesis and enables tumor cells to survive in the presence of elevated ROS (91). Recent studies demonstrated that genetic deletion of *SLC7A11* induces PDAC cell and tumor ferroptosis, and PDAC cells can use cysteine to synthesize GSH and coenzyme A to down-regulate ferroptosis (92, 93).

NADPH could exert functions in maintaining the content of reduced GSH as a coenzyme of glutathione reductase (GR). Recently, a study indicated that increased fatty acid oxidation (FAO) induced by REDD1 deficiency generates NADPH and GSH, which results in decreased oxidative stress and drives KRAS mutant pancreatic cancer progression (136). Moreover, glutamine can also contribute to the cellular redox homeostasis by generating NADPH in PDAC. Son et al. show that glutamine-derived aspartate is transported into the cytoplasm where it can be converted into oxaloacetate by GOT1. Oxaloacetate is converted to malate by MDH1, and then malate is converted to pyruvate to increase NADPH/NADP^+^ ratio through ME1. Importantly, genetic inhibition of these key metabolic enzymes in this pathway leads to an increase in ROS and a reduction in reduced GSH. Hence, NADPH produced by the unique glutamine metabolism manner is indispensable to maintain redox balance and support PDAC growth (9). Furthermore, emerging studies have provided evidence to focus on the effects of diverse factors regulating the unconventional glutamine metabolic pathway enzymes on pancreatic cancer cell redox balance. A recent study has indicated that upregulation of miR-9-5p leads to a significant decrease of NADPH production and corresponding increase of ROS in pancreatic cancer cells through directly inhibiting GOT1 (87). Moreover, CARM1 has been demonstrated to methylate and inhibit MDH1 on arginine 248, which suppresses glutamine metabolism and sensitizes PDAC cells to oxidative stress (19).

## Therapeutic Strategies for Targeting Amino Acids

Due to its important roles in cancer progression, amino acid metabolism is becoming an increasingly promising target for pancreatic cancer therapy. Moreover, some targeting amino acid metabolism strategies in pancreatic cancer have entered to clinical trials (Table 2). Given that PDAC exhibits the increased dependence on glutamine metabolism, the small molecular inhibitors targeting the initiating enzyme in glutamine metabolism GLS1 such as bis-2-(5-phenylacetamido-1,2,4-thiadiazol-2-yl)ethyl sulfide (BPTES), CB-839, and compound 968 have been actively investigated (8). It is worth noting that although GLS inhibition significantly reduced PDAC cell proliferation in short term assays *in vitro*, there is no significant tumor growth delay in mouse models of PDAC (137). Correspondingly, researchers have investigated the effect of combining GLS inhibition and other treatments on pancreatic cancer growth. In a patient-derived pancreatic orthotopic tumor model, encapsulation of BPTES with BPTES nanoparticles (BPTES-NPs) which improved its solubility and improved drug delivery to the pancreatic tumor, attenuates tumor growth more effectively than unencapsulated BPTES. Furthermore, it was revealed that PDAC cells that survive BPTES-NPs treatment are reliant on glycolysis and glycogen synthesis. Thus, combined metformin and BPTES-NPs treatment resulted in significantly greater tumor growth reduction compared with either drug alone (138). Additionally, concomitant treatment of PDAC with GLS inhibitors and ROS generating agents is further demonstrated. ß-lapachone (ß-lap) could cause tumor-selective ROS formation in an NADPH:quinone oxidoreductase 1 (NQO1)-specific manner. NQO1 is highly expressed in up to 90% of PDAC patient specimens, making NQO1-bioactivatable drugs, such as ß-lap especially noteworthy in targeting PDAC. BPTES pre-treatment sensitized mutant KRAS, NQO1 overexpressing PDAC cells to ß-lap, resulting in redox imbalance, extensive DNA damage and PARP-driven metabolic catastrophe. Moreover, the treatment with the CB-839 plus ß-lap combination in the tumor-bearing mice displayed delayed tumor growth and markedly extended survival (139). L-Buthionine-(S,R)-sulfoximine (BSO) is an inhibitor of γ-glutamylcysteine which is important for GSH synthesis. The dual combination of CB-839 and BSO resulted in decreased PDAC cell proliferation *in vitro* and significant tumor growth inhibition *in vivo* (137). Recently, clinical studies evaluating the combination of CB-839 and chemotherapy or targeted therapy in various solid tumors are recruiting (NCT02861300, NCT03965845, and NCT03875313), and assessing the safety, tolerability and efficacy of CB-839 in pancreatic cancer deserves to be considered.

**Table 2:** Clinical trials targeting amino acids metabolism in pancreatic cancer.

**NCT number**	**Status**	**Phase**	**Tumor types**	**Interventions**
**TARGET: ASPARAGINE**				
NCT01523808	Completed	I	Pancreatic cancer	GRASPA
NCT02195180	Completed	II	Metastatic pancreatic adenocarcinoma	ERY001 + Gemcitabine or FOLFOX
NCT03665441	Recruiting	III	Pancreatic adenocarcinoma	Eryaspase + Gemcitabine + Abraxane or Irinotecan + 5-FU + Leucovorin
**TARGET: ARGININE**				
NCT02101580	Terminated	I	Advanced pancreatic cancer	ADI-PEG 20 + Nab-paclitaxel + Gemcitabine
**TARGET: IDO**				
NCT00739609	Terminated	I	Breast Cancer Lung Cancer Melanoma Pancreatic cancer Solid tumors	Indoximod
NCT02077881	Completed	I/II	Metastatic pancreatic adenocarcinoma Metastatic pancreatic cancer	Indoximod + Gemcitabine + Nab-paclitaxel
NCT03432676	Withdrawn	II	Pancreatic ductal adenocarcinoma Stage II pancreatic cancer AJCC v8 Stage IIA pancreatic cancer AJCC v8 Stage IIB pancreatic cancer AJCC v8 Stage III pancreatic cancer AJCC v8 Stage IV pancreatic cancer AJCC v8	Epacadostat + Pembrolizumab
NCT03006302	Recruiting	II	Metastatic pancreatic adenocarcinoma	Epacadostat + Pembrolizumab + CRS-207 ± Cyclophosphamide/GVAX
NCT03085914	Active, not recruiting	I/II	Solid tumors Colorectal cancer Pancreatic ductal adenocarcinoma Lung cancer Urothelial cancer Head and neck cancer	Epacadostat + Pembrolizumab + Chemotherapy^*^

* *Chemotherapy (NCT03085914) is grouped into A (Epacadostat + Pembrolizumab + mFOLFOX6), B (Epacadostat + Pembrolizumab + Gemcitabine + Nab-paclitaxel), C (Epacadostat + Pembrolizumab + Carboplatin + Paclitaxel), D (Epacadostat + Pembrolizumab + Pemetrexed + Carboplatin/Cisplatin), E (Epacadostat + Pembrolizumab + Cyclophosphamide), F (Epacadostat + Pembrolizumab + Gemcitabine + Carboplatin/Cisplatin), and G (Epacadostat + Pembrolizumab + 5-Fluorouracil + Carboplatin/Cisplatin)*.

Apart from glutamine, asparagine is also a critical amino acid for cancer cell survival and growth. Normal cells can receive asparagine via circulating asparagine supply or biosynthesis of asparagine from aspartate and glutamine by ASNS. A large body of evidence has indicated that acute lymphoblastic leukemia (ALL) cells with ASNS deficiency are particularly sensitive to asparagine limitation via L-asparaginase (ASNase) treatment (140). Moreover, emerging studies suggest that the expression of ASNS is downregulated in more than half of PDAC and ASNase treatment could be effective against PDAC growth (141). It has been shown that combined L-asparaginase and general control non-derepressible 2 (GCN2) inhibitor GCN2iA/B or MEK inhibitor PD-325901 could enhance the inhibition of pancreatic cell proliferation and tumor growth (142, 143). In a phase I clinical trial (NCT01523808), asparaginase encapsulated in erythrocytes (ERY-ASP) was well-tolerated by patients with metastatic PDAC (144). Recently, a completed phase IIb clinical trial (NCT02195180) exploring efficacy and safety of ERY-ASP in combination with chemotherapeutic drugs gemcitabine or FOLFOX displayed clinical benefit associated with improvements in overall survival (OS) and progression-free survival (PFS) irrespective of ASNS expression when used in the second-line treatment of advanced pancreatic cancer (145) (Table 2).

Arginine is important for metabolic functions of PDAC including synthesis of other amino acids, proteins, polyamines, and NO. Physiologically, arginine can be synthesized intracellularly from aspartate and citrulline by argininosuccinate synthetase (ASS) and following argininosuccinate lyase (ASL) in the urea cycle. Like asparagine metabolism, reduced ASS expression occurs in pancreatic cancer and ASS-deficient pancreatic cancer exhibits cells and tumor growth inhibition with arginine deprivation achieved by pegylated arginine deiminase (PEG-ADI) treatment (146). Mounting studies suggest that concurrent treatment with PEG-ADI and other drugs is a promising therapeutic strategy for treating ASS-low PDAC. Kim et al. recently discovered that the histone deacetylase (HDAC) inhibitor panobinostat is synergistically lethal with ADI-PEG20 in ASS1-low pancreatic cancer (147). Furthermore, the effect of treating ASS-negative PDAC with the combination of PEG-ADI with chemotherapy or radiotherapy has been well-demonstrated. The combination of PEG-ADI with gemcitabine displayed significant anti-tumor effects in an ASS-deficient PDAC mouse model through a mechanism by which PEG-ADI blocks gemcitabine-mediated overexpression of ribonucleotide reductase subunit M2 (RRM2) through abrogation of the inhibitory effect on E2F-1 activity following gemcitabine exposure (148). In addition, ASS1-deficient pancreatic cancer cells with ADI-PEG20 and docetaxel resulted in translocation of stabilized c-Myc to the nucleus and subsequent increase of hENT1 cell surface expression, which potentiated the effect of gemcitabine treatment via the increase in gemcitabine uptake and provided valuable evidence of combining ADI-PEG20, gemcitabine, and docetaxel for treating ASS1-negative pancreatic cancer (149). A phase 1/1B trial (NCT02101580) evaluating ADI-PEG20 in combination with gemcitabine and nab-paclitaxel in PDAC has been demonstrated that the combination was well-tolerated in some patients with advanced pancreatic cancer, and a further phase 2 trial is under discussion (150) (Table 2). Moreover, ADI-PEG20 enhanced radiation-mediated apoptosis by triggering the ER stress pathway and sensitized ASS1-deficient pancreatic cancer to radiation both *in vitro* and *in vivo* (151). Based on the findings, clinical trials assessing combination of radiation therapy and ADI-PEG20 in ASS1-deficient pancreatic cancer patients deserve to be considered.

In recent years, exploring the association between tumor metabolism and immunity has attracted broad attention and immunotherapy is emerging as a potential therapeutic tool for pancreatic cancer. Pancreatic cancer is characterized by a markedly immunosuppressive microenvironment mediated by immune suppressor cells including tumor associated macrophages (TAMs), regulatory T cells (Tregs) and myeloid-derived suppressor cells (MDSCs), which contributes to tumor progression and metastasis (152). The immunosuppressive activity of MDSCs is associated with the arginine metabolism. MDSCs expressing high levels of arginase and iNOS exhibit inhibition of T cells functions through suppressing T cells proliferation and inducing T cells apoptosis via arginine depletion and NO generation (153). Human MDSCs can be characterized with two main subsets: monocyte-like MDSCs (mMDSCs) and neutrophil-like MDSCs (nMDSCs). In tumor tissues of PDAC patients, nMDSCs, but not mMDSCs, were found to be significantly increased and arginase 1 (ARG1) was predominantly expressed by nMDSCs (154). Further observation discovered that CD13 high nMDSCs expressed higher levels of ARG1 than CD13 low nMDSCs, which endowed CD13 high nMDSCs with stronger immunosuppressive ability (155). Given the high MDSCs heterogeneity, the immune suppressive factor arginase has been a potential target in cancer immunotherapy. Currently, clinical trials evaluating the anti-tumor effect of arginase inhibitor INCB001158 in combination with chemotherapy or immune checkpoint therapeutic agent pembrolizumab in patients with solid tumors are recruiting (NCT03314935 and NCT02903914).

Indoleamine 2,3-dioxygenase (IDO), the rate-limiting enzyme in converting EAA tryptophan to kynurenine, exhibits an immunosuppressive effect in cancer cells. Its role in immunosuppression involves the suppression of CD8+ T effector cells and natural killer cells as well as induction of Tregs and MDSCs (156). Overexpression of IDO has been identified to be associated with poor prognosis in many cancer types including pancreatic cancer (157, 158). Notably, simultaneously targeting IDO and tumor desmoplasia effectively controls tumor growth in mouse models of advanced pancreatic cancer (159). Recently, small molecule inhibitors of IDO such as indoximod, epacadostat, and navoximod are emerging as a therapeutic target in cancer and have been evaluated in clinical trials (160). In pancreatic cancer, a phase I/II clinical trial combining indoximod and chemotherapy was completed (NCT02077881). Moreover, emerging trials evaluating combination of epacadostat, immunotherapy and other therapeutic approaches such as GVAX pancreas vaccine and chemotherapy are ongoing (NCT03006302 and NCT03085914) (Table 2).

## Conclusions and Future Perspectives

Since Otto Warburg made a pioneering discovery on aerobic glycolysis in 1920, mounting studies exploring cancer metabolism have provided substantial opportunities for treating the disease in a century. Cancer cells often exhibit metabolic reprogramming to sustain survival and promote tumor progression even under the harsh environment. The rewiring amino acid metabolism driven by oncogenic factors such as KRAS and MYC as well as tumor suppressors contributes to pancreatic cancer cell growth, invasion, metastasis, angiogenesis, and redox balance. In the hypoxic and nutrient-limiting TME, PDAC has the ability to utilize salvage processes including autophagy and macropinocytosis and reciprocal interaction with stromal components to fuel raising metabolic demand, which is required to sustain tumor growth.

Recently, accumulating evidence has investigated the role of the connection between cellular metabolism especially in amino acids metabolism and epigenetic modifications in cancer cell behavior. On the one hand, changes in tumor cell amino acids metabolism can impact epigenetic regulation. It has been shown that low glutamine in melanoma cells resulted in cancer cell dedifferentiation via histone hypermethylation (161). Serine can contribute to the conversion of methionine to SAM and subsequent DNA and RNA methylation through *de novo* ATP synthesis in colorectal cancer (CRC) cells (162). On the other hand, epigenetic program could in turn alter metabolism in cancer. Histone H3 lysine 9 methyltransferase G9A has been demonstrated to enhance the survival and proliferation of various cancer cells via activation of the serine-glycine biosynthesis pathway (163). In particular, a reciprocal regulation of amino acid import and epigenetic state through a Lat1-EZH2 positive feedback loop in lung cancer has been shown (164). In pancreatic cancer, it has been indicated that LKB1 loss can link serine metabolism to DNA methylation and tumorigenesis (12). Hence, a further understanding the crosstalk between the metabolic and epigenetic rewiring in pancreatic cancer is in high demand and helps to develop promising anti-cancer therapy.

It should be noteworthy that although targeting specific metabolic pathways is effective *in vitro*, the relevant applications *in vivo* may not show the same outcomes due to metabolic plasticity of pancreatic cancer (165). PDAC tumor cells represent a series of metabolic adaptations in resistance to one metabolic perturbation and utilize multiple available nutrients sources and diverse compensatory pathways to maintain growth in the unique TME. Therefore, combination therapies involving targeting adaptive metabolic pathways in PDAC may be a promising approach and evaluating emerging favorable pre-clinical combining strategies in patients can accelerate clinical application. In conclusion, more research is required to investigate the energy metabolism reprogramming in pancreatic cancer, which will develop efficacious therapeutics for treating the deadly disease.

## Author Contributions

YZ and LY designed the study. RX and JY drafted the manuscript. BR, HW, GY, and YC made critical revisions to this manuscript. All authors read and approved the final manuscript.

## Conflict of Interest

The authors declare that the research was conducted in the absence of any commercial or financial relationships that could be construed as a potential conflict of interest.

## Abbreviations

ACC: Acetyl-CoA carboxylases
ACLY: ATP citrate lyase
ALL: Acute lymphoblastic leukemia
AMPA: α-amino-3-hydroxy-5-methyl-4-isoxazolepropionate
AMPARs: α-amino-3-hydroxy-5-methyl-4-isoxazolepropionate receptors
AMPK: AMP-activated protein kinase
ARG2: Arginase 2
ASL: Argininosuccinate lyase
ASNase: L-asparaginase
ASNS: Asparagine synthetase
ASS: Argininosuccinate synthetase
ATF4: Activating transcription factor 4
BCAAs: Branched-chain amino acids
BCAT1: Branched-chain amino acid transaminase 1
BCAT2: Branched-chain amino acid transaminase 2
BCKAs: Branched-chain α-keto acids
BCKDHA: Branched-chain α-keto acid dehydrogenase a
BPTES: Bis-2-(5-phenylacetamido-1,2,4-thiadiazol-2-yl)ethyl sulfide
BPTES-NPs: Bis-2-(5-phenylacetamido-1,2,4-thiadiazol-2-yl)ethyl sulfide nanoparticles
BSO: L-Buthionine-(S,R)-sulfoximine
CAFs: Cancer-associated fibroblasts
CARM1: Coactivator-associated arginine methyltransferase 1
CNS: Central nervous system
CRC: Colorectal cancer
CREB2: cAMP-responsive element binding 2
CSCs: Cancer stem cells
DNMT: DNA methyltransferase
EAAs: Essential amino acids
ECs: Endothelial cells
EDD: E3 ubiquitin ligase identified by differential display
EMT: Epithelial to mesenchymal transition
EMT-TFs: EMT-inducing transcription factors
eNOS: Endothelial nitric oxide synthase
ER: Endoplasmic reticulum
ERY-ASP: Asparaginase encapsulated in erythrocytes
FAO: Fatty acid oxidation
FASN: Fatty acid synthase
FOXO3: Forkhead box transcription factor O 3
GABA: γ-aminobutyric acid
GABRP: γ-aminobutyric acid type A receptor pi subunit
GAD: Glutamic acid decarboxylase
GCL: Glutamate cysteine ligase
GCN2: General control non-derepressible 2
GEMMs: Genetically engineered mouse models
GLS: Glutaminase
GLUD: Glutamate dehydrogenase
GLUL: Glutamate ammonia ligase
GOT: Glutamate-oxaloacetate transaminase
GPT: Glutamate-pyruvate transaminase
GR: Glutathione reductase
GS: Glutathione synthetase
GSH: Glutathione
GSSG: Glutathione disulfide
HDAC: Histone deacetylase
HIF: Hypoxia-induced factor
HO-1: Heme oxygenase-1
HUVECs: Human umbilical vein endothelial cells
IDH1: Isocitrate dehydrogenase 1
IDO: Indoleamine 2,3-dioxygenase
iGluRs: Ionotropic glutamate receptors
IKKβ: I-kappa-B-kinase β
iNOS: Inducible nitric oxide synthase
IPMN: Intraductal papillary mucinous neoplasm
KARs: Kainate receptors
lncRNA: Long non-coding RNA
MDH1: Malate dehydrogenase 1
ME1: Malic enzyme 1
ME2: Malic enzyme 2
mGluRs: Metabotropic glutamate receptors
MMPs: Matrix metalloproteinases
mTOR: Mammalian target of rapamycin
mTORC1: Mammalian target of rapamycin complex 1
mutp53: Mutant p53
NAD: Nicotinamide adenine dinucleotide
NEAAs: Non-essential amino acids
NF-κB: Nuclear factor κB
NMDA: N-methyl-D-aspartate
NMDARs: N-methyl-D-aspartate receptors
nNOS: Neuronal nitric oxide synthase
NO: Nitric oxide
NOS: Nitric oxide synthase
NQO1: NADPH:quinone oxidoreductase 1
NSCLC: Non-small cell lung carcinoma
OAA: Oxaloacetate
OS: Overall survival
P5C: Δ^1^-pyrroline-5-carboxylate
P5CDH: Δ^1^-pyrroline-5-carboxylate dehydrogenase
P5CS: P5C synthase
PanIN: Pancreatic intraepithelial neoplasia
PDAC: Pancreatic ductal adenocarcinoma
PDH: Pyruvate dehydrogenase
PEG-ADI: Pegylated arginine deiminase
PFS: Progression-free survival
PNET: Pancreatic neuroendocrine tumor
POX: Proline oxidase
PP2A: Protein phosphatase 2A
PPARγ: Peroxisome proliferator-activated receptor gamma
PRODH: Proline dehydrogenase
PSCs: Pancreatic stellate cells
PYCR: P5C reductase
ROS: Reactive oxygen species
RRM2: Ribonucleotide reductase subunit M2
SAM: S-adenosyl methionine
SREBP1: Sterol regulatory element-binding protein 1
ß-lap: ß-lapachone
SYK: Spleen tyrosine kinase
TCA: Tricarboxylic acid
TME: Tumor microenvironment
TRIM21: Tripartite-motif-containing protein 21
VEGF: Vascular endothelial growth factor
VEGFR-2: Vascular endothelial growth factor receptor 2
α-KG: α-ketoglutarate.

## References

[1] Siegel RL Miller KD Jemal A. Cancer statistics, 2020. CA Cancer J Clin. (2020) 70:7–30. 10.3322/caac.21590 31912902

[2] Bray F Ferlay J Soerjomataram I Siegel RL Torre LA Jemal A. Global cancer statistics 2018: GLOBOCAN estimates of incidence and mortality worldwide for 36 cancers in 185 countries. CA Cancer J Clin. (2018) 68:394–424. 10.3322/caac.21492 30207593

[3] Neoptolemos JP Kleeff J Michl P Costello E Greenhalf W Palmer DH. Therapeutic developments in pancreatic cancer: current and future perspectives. Nat Rev Gastroenterol Hepatol. (2018) 15:333–48. 10.1038/s41575-018-0005-x 29717230

[4] Hanahan D Weinberg RA. Hallmarks of cancer: the next generation. Cell. (2011) 144:646–74. 10.1016/j.cell.2011.02.013 21376230

[5] Yang J Ren B Yang G Wang H Chen G You L. The enhancement of glycolysis regulates pancreatic cancer metastasis. Cell Mol Life Sci. (2020) 77:305–21. 10.1007/s00018-019-03278-z 31432232 PMC11104916

[6] Li J Gu D Lee SSY Song B Bandyopadhyay S Chen S. Abrogating cholesterol esterification suppresses growth and metastasis of pancreatic cancer. Oncogene. (2016) 35:6378–88. 10.1038/onc.2016.168 27132508 PMC5093084

[7] Reeds PJ. Dispensable and indispensable amino acids for humans. J Nutr. (2000) 130:1835S−40S. 10.1093/jn/130.7.1835S 10867060

[8] Altman BJ Stine ZE Dang CV. From Krebs to clinic: glutamine metabolism to cancer therapy. Nat Rev Cancer. (2016) 16:619–34. 10.1038/nrc.2016.71 27492215 PMC5484415

[9] Son J Lyssiotis CA Ying H Wang X Hua S Ligorio M. Glutamine supports pancreatic cancer growth through a KRAS-regulated metabolic pathway. Nature. (2013) 496:101–5. 10.1038/nature12040 23535601 PMC3656466

[10] Gao X Sanderson SM Dai Z Reid MA Cooper DE Lu M. Dietary methionine influences therapy in mouse cancer models and alters human metabolism. Nature. (2019) 572:397–401. 10.1038/s41586-019-1437-3 31367041 PMC6951023

[11] Sousa CM Biancur DE Wang X Halbrook CJ Sherman MH Zhang L. Pancreatic stellate cells support tumour metabolism through autophagic alanine secretion. Nature. (2016) 536:479–83. 10.1038/nature19084 27509858 PMC5228623

[12] Kottakis F Nicolay BN Roumane A Karnik R Gu H Nagle JM. LKB1 loss links serine metabolism to DNA methylation and tumorigenesis. Nature. (2016) 539:390–5. 10.1038/nature20132 27799657 PMC5988435

[13] Vernieri C Casola S Foiani M Pietrantonio F de Braud F Longo V. Targeting cancer metabolism: dietary and pharmacologic interventions. Cancer Discov. (2016) 6:1315–33. 10.1158/2159-8290.CD-16-0615 27872127 PMC5140697

[14] Kleeff J Korc M Apte M La Vecchia C Johnson CD Biankin AV. Pancreatic cancer. Nat Rev Dis Primers. (2016) 2:16022. 10.1038/nrdp.2016.22 27158978

[15] Ying H Dey P Yao W Kimmelman AC Draetta GF Maitra A. Genetics and biology of pancreatic ductal adenocarcinoma. Genes Dev. (2016) 30:355–85. 10.1101/gad.275776.115 26883357 PMC4762423

[16] Jones S Zhang X Parsons DW Lin JC-H Leary RJ Angenendt P. Core signaling pathways in human pancreatic cancers revealed by global genomic analyses. Science. (2008) 321:1801–6. 10.1126/science.1164368 18772397 PMC2848990

[17] Waddell N Pajic M Patch A-M Chang DK Kassahn KS Bailey P. Whole genomes redefine the mutational landscape of pancreatic cancer. Nature. (2015) 518:495–501. 10.1038/nature14169 25719666 PMC4523082

[18] Pylayeva-Gupta Y Grabocka E Bar-Sagi D. RAS oncogenes: weaving a tumorigenic web. Nat Rev Cancer. (2011) 11:761–74. 10.1038/nrc3106 21993244 PMC3632399

[19] Wang Y-P Zhou W Wang J Huang X Zuo Y Wang T-S. Arginine methylation of mdh1 by carm1 inhibits glutamine metabolism and suppresses pancreatic cancer. Mol Cell. (2016) 64:673–87. 10.1016/j.molcel.2016.09.028 27840030

[20] Mukhopadhyay S Goswami D Adiseshaiah PP Burgan W Yi M Guerin TM. Undermining glutaminolysis bolsters chemotherapy while NRF2 promotes chemoresistance in KRAS-driven pancreatic cancers. Cancer research. (2020) 80:1630–43. 10.1158/0008-5472.CAN-19-1363 31911550 PMC7185043

[21] Sivanand S Vander Heiden MG. Emerging roles for branched-chain amino acid metabolism in cancer. Cancer Cell. (2020) 37:147–56. 10.1016/j.ccell.2019.12.011 32049045 PMC7082774

[22] Mayers JR Wu C Clish CB Kraft P Torrence ME Fiske BP. Elevation of circulating branched-chain amino acids is an early event in human pancreatic adenocarcinoma development. Nat Med. (2014) 20:1193–8. 10.1038/nm.3686 25261994 PMC4191991

[23] Li J-T Yin M Wang D Wang J Lei M-Z Zhang Y. BCAT2-mediated BCAA catabolism is critical for development of pancreatic ductal adenocarcinoma. Nature Cell Biol. (2020) 22:167–74. 10.1038/s41556-019-0455-6 32029896

[24] Gabay M Li Y Felsher DW. MYC activation is a hallmark of cancer initiation and maintenance. Cold Spring Harb Perspect Med. (2014) 4:a014241. 10.1101/cshperspect.a014241 24890832 PMC4031954

[25] Dang CV Le A Gao P. MYC-induced cancer cell energy metabolism and therapeutic opportunities. Clin Cancer Res. (2009) 15:6479–83. 10.1158/1078-0432.CCR-09-0889 19861459 PMC2783410

[26] Hessmann E Schneider G Ellenrieder V Siveke JT. MYC in pancreatic cancer: novel mechanistic insights and their translation into therapeutic strategies. Oncogene. (2016) 35:1609–18. 10.1038/onc.2015.216 26119937

[27] Wise DR DeBerardinis RJ Mancuso A Sayed N Zhang X-Y Pfeiffer HK. Myc regulates a transcriptional program that stimulates mitochondrial glutaminolysis and leads to glutamine addiction. Proc Natl Acad Sci USA. (2008) 105:18782–7. 10.1073/pnas.0810199105 19033189 PMC2596212

[28] Gao P Tchernyshyov I Chang T-C Lee Y-S Kita K Ochi T. c-Myc suppression of miR-23a/b enhances mitochondrial glutaminase expression and glutamine metabolism. Nature. (2009) 458:762–5. 10.1038/nature07823 19219026 PMC2729443

[29] Csibi A Lee G Yoon S-O Tong H Ilter D Elia I. The mTORC1/S6K1 pathway regulates glutamine metabolism through the eIF4B-dependent control of c-Myc translation. Curr Biol. (2014) 24:2274–80. 10.1016/j.cub.2014.08.007 25220053 PMC4190129

[30] Deng S-J Chen H-Y Zeng Z Deng S Zhu S Ye Z. Nutrient stress-dysregulated antisense lncRNA GLS-AS impairs GLS-mediated metabolism and represses pancreatic cancer progression. Cancer Res. (2019) 79:1398–412. 10.1158/0008-5472.CAN-18-0419 30563888

[31] He J Li F Zhou Y Hou X Liu S Li X. LncRNA XLOC_006390 promotes pancreatic carcinogenesis and glutamate metabolism by stabilizing c-Myc. Cancer Lett. (2020) 469:419–28. 10.1016/j.canlet.2019.11.021 31734356

[32] Liu W Le A Hancock C Lane AN Dang CV Fan TWM. Reprogramming of proline and glutamine metabolism contributes to the proliferative and metabolic responses regulated by oncogenic transcription factor c-MYC. Proc Natl Acad Sci USA. (2012) 109:8983–8. 10.1073/pnas.1203244109 22615405 PMC3384197

[33] Bieging KT Mello SS Attardi LD. Unravelling mechanisms of p53-mediated tumour suppression. Nat Rev Cancer. (2014) 14:359–70. 10.1038/nrc3711 24739573 PMC4049238

[34] Cheung EC Vousden KH. The role of p53 in glucose metabolism. Curr Opin Cell Biol. (2010) 22:186–91. 10.1016/j.ceb.2009.12.006 20061129

[35] Suzuki S Tanaka T Poyurovsky MV Nagano H Mayama T Ohkubo S. Phosphate-activated glutaminase (GLS2), a p53-inducible regulator of glutamine metabolism and reactive oxygen species. Proc Natl Acad Sci USA. (2010) 107:7461–6. 10.1073/pnas.1002459107 20351271 PMC2867754

[36] Hu W Zhang C Wu R Sun Y Levine A Feng Z. Glutaminase 2, a novel p53 target gene regulating energy metabolism and antioxidant function. Proc Natl Acad Sci USA. (2010) 107:7455–60. 10.1073/pnas.1001006107 20378837 PMC2867677

[37] Kruiswijk F Labuschagne CF Vousden KH. p53 in survival, death and metabolic health: a lifeguard with a licence to kill. Nat Rev Mol Cell Biol. (2015) 16:393–405. 10.1038/nrm4007 26122615

[38] Reid MA Wang W-I Rosales KR Welliver MX Pan M Kong M. The B55α subunit of PP2A drives a p53-dependent metabolic adaptation to glutamine deprivation. Mol Cell. (2013) 50:200–11. 10.1016/j.molcel.2013.02.008 23499005

[39] Ishak Gabra MB Yang Y Lowman XH Reid MA Tran TQ Kong M. IKKβ activates p53 to promote cancer cell adaptation to glutamine deprivation. Oncogenesis. (2018) 7:93. 10.1038/s41389-018-0104-0 30478303 PMC6255781

[40] Lowman XH Hanse EA Yang Y Ishak Gabra MB Tran TQ Li H. p53 promotes cancer cell adaptation to glutamine deprivation by upregulating slc7a3 to increase arginine uptake. Cell Rep. (2019) 26:3051–60.e4. 10.1016/j.celrep.2019.02.037 30865893 PMC6510239

[41] Tajan M Hock AK Blagih J Robertson NA Labuschagne CF Kruiswijk F. A role for p53 in the adaptation to glutamine starvation through the expression of SLC1A3. Cell Metab. (2018) 28:721–36.e6. 10.1016/j.cmet.2018.07.005 30122553 PMC6224545

[42] Muller PAJ Vousden KH. p53 mutations in cancer. Nat Cell Biol. (2013) 15:2–8. 10.1038/ncb2641 23263379

[43] Tran TQ Lowman XH Reid MA Mendez-Dorantes C Pan M Yang Y. Tumor-associated mutant p53 promotes cancer cell survival upon glutamine deprivation through p21 induction. Oncogene. (2017) 36:1991–2001. 10.1038/onc.2016.360 27721412 PMC5383530

[44] Maddocks ODK Berkers CR Mason SM Zheng L Blyth K Gottlieb E. Serine starvation induces stress and p53-dependent metabolic remodelling in cancer cells. Nature. (2013) 493:542–6. 10.1038/nature11743 23242140 PMC6485472

[45] Haigis MC Mostoslavsky R Haigis KM Fahie K Christodoulou DC Murphy AJ. SIRT4 inhibits glutamate dehydrogenase and opposes the effects of calorie restriction in pancreatic beta cells. Cell. (2006) 126:941–54. 10.1016/j.cell.2006.06.057 16959573

[46] Csibi A Fendt S-M Li C Poulogiannis G Choo AY Chapski DJ. The mTORC1 pathway stimulates glutamine metabolism and cell proliferation by repressing SIRT4. Cell. (2013) 153:840–54. 10.1016/j.cell.2013.04.023 23663782 PMC3684628

[47] Jeong SM Xiao C Finley LWS Lahusen T Souza AL Pierce K. SIRT4 has tumor-suppressive activity and regulates the cellular metabolic response to DNA damage by inhibiting mitochondrial glutamine metabolism. Cancer Cell. (2013) 23:450–63. 10.1016/j.ccr.2013.02.024 23562301 PMC3650305

[48] Hu Q Qin Y Ji S Xu W Liu W Sun Q. UHRF1 promotes aerobic glycolysis and proliferation via suppression of SIRT4 in pancreatic cancer. Cancer Lett. (2019) 452:226–36. 10.1016/j.canlet.2019.03.024 30905812

[49] Dey P Baddour J Muller F Wu CC Wang H Liao W-T. Genomic deletion of malic enzyme 2 confers collateral lethality in pancreatic cancer. Nature. (2017) 542:119–23. 10.1038/nature21052 28099419 PMC5398413

[50] Mayers JR Torrence ME Danai LV Papagiannakopoulos T Davidson SM Bauer MR. Tissue of origin dictates branched-chain amino acid metabolism in mutant Kras-driven cancers. Science. (2016) 353:1161–5. 10.1126/science.aaf5171 27609895 PMC5245791

[51] Pandhare J Cooper SK Phang JM. Proline oxidase, a proapoptotic gene, is induced by troglitazone: evidence for both peroxisome proliferator-activated receptor gamma-dependent and -independent mechanisms. J Biol Chem. (2006) 281:2044–52. 10.1074/jbc.M507867200 16303758

[52] Liu W Glunde K Bhujwalla ZM Raman V Sharma A Phang JM. Proline oxidase promotes tumor cell survival in hypoxic tumor microenvironments. Cancer Res. (2012) 72:3677–86. 10.1158/0008-5472.CAN-12-0080 22609800 PMC3399032

[53] Guillaumond F Leca J Olivares O Lavaut M-N Vidal N Berthezène P. Strengthened glycolysis under hypoxia supports tumor symbiosis and hexosamine biosynthesis in pancreatic adenocarcinoma. Proc Natl Acad Sci USA. (2013) 110:3919–24. 10.1073/pnas.1219555110 23407165 PMC3593894

[54] He G Jiang Y Zhang B Wu G. The effect of HIF-1α on glucose metabolism, growth and apoptosis of pancreatic cancerous cells. Asia Pac J Clin Nutr. (2014) 23:174–80. 24561986 10.6133/apjcn.2014.23.1.14

[55] Zhang Q Lou Y Zhang J Fu Q Wei T Sun X. Hypoxia-inducible factor-2α promotes tumor progression and has crosstalk with Wnt/β-catenin signaling in pancreatic cancer. Mol Cancer. (2017) 16:119. 10.1186/s12943-017-0689-5 28705232 PMC5512744

[56] Li W Chen C Zhao X Ye H Zhao Y Fu Z. HIF-2α regulates non-canonical glutamine metabolism via activation of PI3K/mTORC2 pathway in human pancreatic ductal adenocarcinoma. J Cell Mol Med. (2017) 21:2896–908. 10.1111/jcmm.13202 28544376 PMC5661146

[57] Yoo HC Park SJ Nam M Kang J Kim K Yeo JH. A variant of SLC1A5 is a mitochondrial glutamine transporter for metabolic reprogramming in cancer cells. Cell Metab. (2020) 31:267–83.e12. 10.1016/j.cmet.2019.11.020 31866442

[58] Halbrook CJ Lyssiotis CA. Employing metabolism to improve the diagnosis and treatment of pancreatic cancer. Cancer Cell. (2017) 31:5–19. 10.1016/j.ccell.2016.12.006 28073003

[59] Fu Y Liu S Zeng S Shen H. The critical roles of activated stellate cells-mediated paracrine signaling, metabolism and onco-immunology in pancreatic ductal adenocarcinoma. Mol Cancer. (2018) 17:62. 10.1186/s12943-018-0815-z 29458370 PMC5817854

[60] Richards KE Zeleniak AE Fishel ML Wu J Littlepage LE Hill R. Cancer-associated fibroblast exosomes regulate survival and proliferation of pancreatic cancer cells. Oncogene. (2017) 36:1770–8. 10.1038/onc.2016.353 27669441 PMC5366272

[61] Zhao H Yang L Baddour J Achreja A Bernard V Moss T. Tumor microenvironment derived exosomes pleiotropically modulate cancer cell metabolism. Elife. (2016) 5:e10250. 10.7554/eLife.10250 26920219 PMC4841778

[62] Kreso A Dick JE. Evolution of the cancer stem cell model. Cell Stem Cell. (2014) 14:275–91. 10.1016/j.stem.2014.02.006 24607403

[63] Wang VMY Ferreira RMM Almagro J Evan T Legrave N Zaw Thin M. CD9 identifies pancreatic cancer stem cells and modulates glutamine metabolism to fuel tumour growth. Nat Cell Biol. (2019) 21:1425–35. 10.1038/s41556-019-0407-1 31685994 PMC6944508

[64] Zyromski NJ Mathur A Pitt HA Wade TE Wang S Nakshatri P. Obesity potentiates the growth and dissemination of pancreatic cancer. Surgery. (2009) 146:258–63. 10.1016/j.surg.2009.02.024 19628082

[65] Meyer KA Neeley CK Baker NA Washabaugh AR Flesher CG Nelson BS. Adipocytes promote pancreatic cancer cell proliferation via glutamine transfer. Biochem Biophys Rep. (2016) 7:144–9. 10.1016/j.bbrep.2016.06.004 27617308 PMC5014359

[66] Olivares O Mayers JR Gouirand V Torrence ME Gicquel T Borge L. Collagen-derived proline promotes pancreatic ductal adenocarcinoma cell survival under nutrient limited conditions. Nat Commun. (2017) 8:16031. 10.1038/ncomms16031 28685754 PMC5504351

[67] Yang S Wang X Contino G Liesa M Sahin E Ying H. Pancreatic cancers require autophagy for tumor growth. Genes Dev. (2011) 25:717–29. 10.1101/gad.2016111 21406549 PMC3070934

[68] Perera RM Stoykova S Nicolay BN Ross KN Fitamant J Boukhali M. Transcriptional control of autophagy-lysosome function drives pancreatic cancer metabolism. Nature. (2015) 524:361–5. 10.1038/nature14587 26168401 PMC5086585

[69] Bryant KL Stalnecker CA Zeitouni D Klomp JE Peng S Tikunov AP. Combination of ERK and autophagy inhibition as a treatment approach for pancreatic cancer. Nat Med. (2019) 25:628–40. 10.1038/s41591-019-0368-8 30833752 PMC6484853

[70] Commisso C Davidson SM Soydaner-Azeloglu RG Parker SJ Kamphorst JJ Hackett S. Macropinocytosis of protein is an amino acid supply route in Ras-transformed cells. Nature. (2013) 497:633–7. 10.1038/nature12138 23665962 PMC3810415

[71] Kamphorst JJ Nofal M Commisso C Hackett SR Lu W Grabocka E. Human pancreatic cancer tumors are nutrient poor and tumor cells actively scavenge extracellular protein. Cancer Res. (2015) 75:544–53. 10.1158/0008-5472.CAN-14-2211 25644265 PMC4316379

[72] Davidson SM Jonas O Keibler MA Hou HW Luengo A Mayers JR. Direct evidence for cancer-cell-autonomous extracellular protein catabolism in pancreatic tumors. Nat Med. (2017) 23:235–41. 10.1038/nm.4256 28024083 PMC5407288

[73] Mossmann D Park S Hall MN. mTOR signalling and cellular metabolism are mutual determinants in cancer. Nat Rev Cancer. (2018) 18:744–57. 10.1038/s41568-018-0074-8 30425336

[74] Rebsamen M Pochini L Stasyk T de Araújo MEG Galluccio M Kandasamy RK. SLC38A9 is a component of the lysosomal amino acid sensing machinery that controls mTORC1. Nature. (2015) 519:477–81. 10.1038/nature14107 25561175 PMC4376665

[75] Wyant GA Abu-Remaileh M Wolfson RL Chen WW Freinkman E Danai LV. mTORC1 activator SLC38A9 is required to efflux essential amino acids from lysosomes and use protein as a nutrient. Cell. (2017) 171:642–54.e12. 10.1016/j.cell.2017.09.046 29053970 PMC5704964

[76] Palm W Park Y Wright K Pavlova NN Tuveson DA Thompson CB. The utilization of extracellular proteins as nutrients is suppressed by mTORC1. Cell. (2015) 162:259–70. 10.1016/j.cell.2015.06.017 26144316 PMC4506698

[77] He C Klionsky DJ. Regulation mechanisms and signaling pathways of autophagy. Annu Rev Genet. (2009) 43:67–93. 10.1146/annurev-genet-102808-114910 19653858 PMC2831538

[78] Thompson CB Palm W. Reexamining how cancer cells exploit the body's metabolic resources. Cold Spring Harb Symp Quant Biol. (2016) 81:67–72. 10.1101/sqb.2016.81.030734 28396524

[79] Nofal M Zhang K Han S Rabinowitz JD. mTOR inhibition restores amino acid balance in cells dependent on catabolism of extracellular protein. Mol Cell. (2017) 67:936–46.e5. 10.1016/j.molcel.2017.08.011 28918901 PMC5612669

[80] Kanda M Matthaei H Wu J Hong S-M Yu J Borges M. Presence of somatic mutations in most early-stage pancreatic intraepithelial neoplasia. Gastroenterology. (2012) 142:730–33.e9. 10.1053/j.gastro.2011.12.042 22226782 PMC3321090

[81] Collet L Ghurburrun E Meyers N Assi M Pirlot B Leclercq IA. Kras and Lkb1 mutations synergistically induce intraductal papillary mucinous neoplasm derived from pancreatic duct cells. Gut. (2020) 69:704–14. 10.1136/gutjnl-2018-318059 31154393

[82] Kopp JL Dubois CL Schaeffer DF Samani A Taghizadeh F Cowan RW. Loss of Pten and activation of kras synergistically induce formation of intraductal papillary mucinous neoplasia from pancreatic ductal cells in Mice. Gastroenterology. (2018) 154:1509–23.e5. 10.1053/j.gastro.2017.12.007 29273451 PMC5880733

[83] Lampson BL Kendall SD Ancrile BB Morrison MM Shealy MJ Barrientos KS. Targeting eNOS in pancreatic cancer. Cancer Res. (2012) 72:4472–82. 10.1158/0008-5472.CAN-12-0057 22738914 PMC3749841

[84] Bott AJ Shen J Tonelli C Zhan L Sivaram N Jiang Y-P. Glutamine anabolism plays a critical role in pancreatic cancer by coupling carbon and nitrogen metabolism. Cell Rep. (2019) 29:1287–98.e6. 10.1016/j.celrep.2019.09.056 31665640 PMC6886125

[85] Yoshida T Yamasaki S Kaneko O Taoka N Tomimoto Y Namatame I. A covalent small molecule inhibitor of glutamate-oxaloacetate transaminase 1 impairs pancreatic cancer growth. Biochem Biophys Res Commun. (2020) 522:633–8. 10.1016/j.bbrc.2019.11.130 31787239 PMC6981064

[86] Nelson BS Lin L Kremer DM Sousa CM Cotta-Ramusino C Myers A. Tissue of origin dictates GOT1 dependence and confers synthetic lethality to radiotherapy. Cancer Metab. (2020) 8:1. 10.1101/714196 31908776 PMC6941320

[87] Wang J Wang B Ren H Chen W. miR-9-5p inhibits pancreatic cancer cell proliferation, invasion and glutamine metabolism by targeting GOT1. Biochem Biophys Res Commun. (2019) 509:241–8. 10.1016/j.bbrc.2018.12.114 30591220

[88] Yang S Hwang S Kim M Seo SB Lee J-H Jeong SM. Mitochondrial glutamine metabolism via GOT2 supports pancreatic cancer growth through senescence inhibition. Cell Death Dis. (2018) 9:55. 10.1038/s41419-017-0089-1 29352139 PMC5833441

[89] Yang H Zhou L Shi Q Zhao Y Lin H Zhang M. SIRT3-dependent GOT2 acetylation status affects the malate-aspartate NADH shuttle activity and pancreatic tumor growth. EMBO J. (2015) 34:1110–25. 10.15252/embj.201591041 25755250 PMC4406655

[90] Lee JH Cho Y-R Kim JH Kim J Nam HY Kim SW. Branched-chain amino acids sustain pancreatic cancer growth by regulating lipid metabolism. Exp Mol Med. (2019) 51:1–11. 10.1038/s12276-019-0350-z 31784505 PMC6884453

[91] Lo M Ling V Wang YZ Gout PW. The xc- cystine/glutamate antiporter: a mediator of pancreatic cancer growth with a role in drug resistance. Br J Cancer. (2008) 99:464–72. 10.1038/sj.bjc.6604485 18648370 PMC2527809

[92] Daher B Parks SK Durivault J Cormerais Y Baidarjad H Tambutte E. Genetic ablation of the cystine transporter xCT in PDAC cells inhibits mTORC1, growth, survival, and tumor formation via nutrient and oxidative stresses. Cancer Res. (2019) 79:3877–90. 10.1158/0008-5472.CAN-18-3855 31175120

[93] Badgley MA Kremer DM Maurer HC DelGiorno KE Lee H-J Purohit V. Cysteine depletion induces pancreatic tumor ferroptosis in mice. Science. (2020) 368:85–9. 10.1126/science.aaw9872 32241947 PMC7681911

[94] Zaytouni T Tsai P-Y Hitchcock DS DuBois CD Freinkman E Lin L. Critical role for arginase 2 in obesity-associated pancreatic cancer. Nat Commun. (2017) 8:242. 10.1038/s41467-017-00331-y 28808255 PMC5556090

[95] Takehara A Hosokawa M Eguchi H Ohigashi H Ishikawa O Nakamura Y. Gamma-aminobutyric acid (GABA) stimulates pancreatic cancer growth through overexpressing GABAA receptor pi subunit. Cancer Res. (2007) 67:9704–12. 10.1158/0008-5472.CAN-07-2099 17942900

[96] Gaianigo N Melisi D Carbone C. EMT and treatment resistance in pancreatic cancer. Cancers. (2017) 9:1046–9. 10.3390/cancers9090122 28895920 PMC5615337

[97] Rhim AD Mirek ET Aiello NM Maitra A Bailey JM McAllister F. EMT and dissemination precede pancreatic tumor formation. Cell. (2012) 148:349–61. 10.1016/j.cell.2011.11.025 22265420 PMC3266542

[98] De Craene B Berx G. Regulatory networks defining EMT during cancer initiation and progression. Nat Rev Cancer. (2013) 13:97–100. 10.1038/nrc3447 23344542

[99] Lambert AW Pattabiraman DR Weinberg RA. Emerging biological principles of metastasis. Cell. (2017) 168:670–91. 10.1016/j.cell.2016.11.037 28187288 PMC5308465

[100] Zheng X Carstens JL Kim J Scheible M Kaye J Sugimoto H. Epithelial-to-mesenchymal transition is dispensable for metastasis but induces chemoresistance in pancreatic cancer. Nature. (2015) 527:525–30. 10.1038/nature16064 26560028 PMC4849281

[101] Krebs AM Mitschke J Lasierra Losada M Schmalhofer O Boerries M Busch H. The EMT-activator Zeb1 is a key factor for cell plasticity and promotes metastasis in pancreatic cancer. Nat Cell Biol. (2017) 19:518–29. 10.1038/ncb3513 28414315

[102] Wang H Li Q-F Chow HY Choi SC Leung Y-C. Arginine deprivation inhibits pancreatic cancer cell migration, invasion and EMT via the down regulation of snail, slug, twist, and MMP1/9. J Physiol Biochem. (2020) 76:73–83. 10.1007/s13105-019-00716-1 31823303

[103] Vickers SM MacMillan-Crow LA Green M Ellis C Thompson JA. Association of increased immunostaining for inducible nitric oxide synthase and nitrotyrosine with fibroblast growth factor transformation in pancreatic cancer. Arch Surg. (1999) 134:245–51. 10.1001/archsurg.134.3.245 10088562

[104] Lim K-H Ancrile BB Kashatus DF Counter CM. Tumour maintenance is mediated by eNOS. Nature. (2008) 452:646–9. 10.1038/nature06778 18344980 PMC2688829

[105] Wang J He P Gaida M Yang S Schetter AJ Gaedcke J. Inducible nitric oxide synthase enhances disease aggressiveness in pancreatic cancer. Oncotarget. (2016) 7:52993–3004. 10.18632/oncotarget.10323 27367029 PMC5288163

[106] O'Leary BR Fath MA Bellizzi AM Hrabe JE Button AM Allen BG. Loss of SOD3 (EcSOD) expression promotes an aggressive phenotype in human pancreatic ductal adenocarcinoma. Clin Cancer Res. (2015) 21:1741–51. 10.1158/1078-0432.CCR-14-1959 25634994 PMC4383686

[107] Wang J Yang S He P Schetter AJ Gaedcke J Ghadimi BM. Endothelial Nitric Oxide Synthase Traffic Inducer (NOSTRIN) is a negative regulator of disease aggressiveness in pancreatic cancer. Clin Cancer Res. (2016) 22:5992–6001. 10.1158/1078-0432.CCR-16-0511 27401251 PMC5161709

[108] Fujita M Imadome K Endo S Shoji Y Yamada S Imai T. Nitric oxide increases the invasion of pancreatic cancer cells via activation of the PI3K-AKT and RhoA pathways after carbon ion irradiation. FEBS Lett. (2014) 588:3240–50. 10.1016/j.febslet.2014.07.006 25019574

[109] Reiner A Levitz J. Glutamatergic signaling in the central nervous system: ionotropic and metabotropic receptors in concert. Neuron. (2018) 98:1080–98. 10.1016/j.neuron.2018.05.018 29953871 PMC6484838

[110] Herner A Sauliunaite D Michalski CW Erkan M De Oliveira T Abiatari I. Glutamate increases pancreatic cancer cell invasion and migration via AMPA receptor activation and Kras-MAPK signaling. Int J Cancer. (2011) 129:2349–59. 10.1002/ijc.25898 21207374

[111] Li L Hanahan D. Hijacking the neuronal NMDAR signaling circuit to promote tumor growth and invasion. Cell. (2013) 153:86–100. 10.1016/j.cell.2013.02.051 23540692

[112] Li L Zeng Q Bhutkar A Galván JA Karamitopoulou E Noordermeer D. GKAP acts as a genetic modulator of NMDAR signaling to govern invasive tumor growth. Cancer Cell. (2018) 33:736–51.e5. 10.1016/j.ccell.2018.02.011 29606348 PMC5896248

[113] Petroff OAC. GABA and glutamate in the human brain. Neuroscientist. (2002) 8:562–73. 10.1177/1073858402238515 12467378

[114] Jiang S-H Hu L-P Wang X Li J Zhang Z-G. Neurotransmitters: emerging targets in cancer. Oncogene. (2020) 39:503–15. 10.1038/s41388-019-1006-0 31527667

[115] Schuller HM Al-Wadei HAN Majidi M. GABA B receptor is a novel drug target for pancreatic cancer. Cancer. (2008) 112:767–78. 10.1002/cncr.23231 18098271 PMC3375598

[116] Jiang S-H Zhu L-L Zhang M Li R-K Yang Q Yan J-Y. GABRP regulates chemokine signalling, macrophage recruitment and tumour progression in pancreatic cancer through tuning KCNN4-mediated Ca signalling in a GABA-independent manner. Gut. (2019) 68:1994–2006. 10.1136/gutjnl-2018-317479 30826748

[117] Li S Xu H-X Wu C-T Wang W-Q Jin W Gao H-L. Angiogenesis in pancreatic cancer: current research status and clinical implications. Angiogenesis. (2019) 22:15–36. 10.1007/s10456-018-9645-2 30168025

[118] Potente M Carmeliet P. The link between angiogenesis and endothelial metabolism. Annu Rev Physiol. (2017) 79:43–66. 10.1146/annurev-physiol-021115-105134 27992732

[119] De Bock K Georgiadou M Schoors S Kuchnio A Wong BW Cantelmo AR. Role of PFKFB3-driven glycolysis in vessel sprouting. Cell. (2013) 154:651–63. 10.1016/j.cell.2013.06.037 23911327

[120] Huang H Vandekeere S Kalucka J Bierhansl L Zecchin A Brüning U. Role of glutamine and interlinked asparagine metabolism in vessel formation. EMBO J. (2017) 36:2334–52. 10.15252/embj.201695518 28659375 PMC5556263

[121] Kim B Li J Jang C Arany Z. Glutamine fuels proliferation but not migration of endothelial cells. EMBO J. (2017) 36:2321–33. 10.15252/embj.201796436 28659379 PMC5556269

[122] Fukumura D Kashiwagi S Jain RK. The role of nitric oxide in tumour progression. Nat Rev Cancer. (2006) 6:521–34. 10.1038/nrc1910 16794635

[123] Kimura H Weisz A Kurashima Y Hashimoto K Ogura T D'Acquisto F. Hypoxia response element of the human vascular endothelial growth factor gene mediates transcriptional regulation by nitric oxide: control of hypoxia-inducible factor-1 activity by nitric oxide. Blood. (2000) 95:189–97. 10.1182/blood.V95.1.189.001k05_189_197 10607702

[124] Pae H-O Oh G-S Choi B-M Kim Y-M Chung H-T. A molecular cascade showing nitric oxide-heme oxygenase-1-vascular endothelial growth factor-interleukin-8 sequence in human endothelial cells. Endocrinology. (2005) 146:2229–38. 10.1210/en.2004-1431 15661856

[125] Papapetropoulos A García-Cardeña G Madri JA Sessa WC. Nitric oxide production contributes to the angiogenic properties of vascular endothelial growth factor in human endothelial cells. J Clin Invest. (1997) 100:3131–9. 10.1172/JCI119868 9399960 PMC508526

[126] Fukumura D Gohongi T Kadambi A Izumi Y Ang J Yun CO. Predominant role of endothelial nitric oxide synthase in vascular endothelial growth factor-induced angiogenesis and vascular permeability. Proc Natl Acad Sci USA. (2001) 98:2604–9. 10.1073/pnas.041359198 11226286 PMC30185

[127] Lala PK Chakraborty C. Role of nitric oxide in carcinogenesis and tumour progression. Lancet Oncol. (2001) 2:149–56. 10.1016/S1470-2045(00)00256-4 11902565

[128] Nussler AK Gansauge S Gansauge F Fischer U Butzer U Kremsner PG. Overexpression of endothelium-derived nitric oxide synthase isoform 3 in the vasculature of human pancreatic tumor biopsies. Langenbecks Arch Surg. (1998) 383:474–80. 10.1007/s004230050163 9921950

[129] Camp ER Yang A Liu W Fan F Somcio R Hicklin DJ. Roles of nitric oxide synthase inhibition and vascular endothelial growth factor receptor-2 inhibition on vascular morphology and function in an in vivo model of pancreatic cancer. Clin Cancer Res. (2006) 12:2628–33. 10.1158/1078-0432.CCR-05-2257 16638876

[130] Gorrini C Harris IS Mak TW. Modulation of oxidative stress as an anticancer strategy. Nat Rev Drug Discov. (2013) 12:931–47. 10.1038/nrd4002 24287781

[131] Chen R Lai LA Sullivan Y Wong M Wang L Riddell J. Disrupting glutamine metabolic pathways to sensitize gemcitabine-resistant pancreatic cancer. Sci Rep. (2017) 7:7950. 10.1038/s41598-017-08436-6 28801576 PMC5554139

[132] Lu SC. Regulation of glutathione synthesis. Mol Aspects Med. (2009) 30:42–59. 10.1016/j.mam.2008.05.005 18601945 PMC2704241

[133] Koppula P Zhang Y Zhuang L Gan B. Amino acid transporter SLC7A11/xCT at the crossroads of regulating redox homeostasis and nutrient dependency of cancer. Cancer Commun. (2018) 38:12. 10.1186/s40880-018-0288-x 29764521 PMC5993148

[134] Timmerman LA Holton T Yuneva M Louie RJ Padró M Daemen A. Glutamine sensitivity analysis identifies the xCT antiporter as a common triple-negative breast tumor therapeutic target. Cancer Cell. (2013) 24:450–65. 10.1016/j.ccr.2013.08.020 24094812 PMC3931310

[135] Yuneva M Zamboni N Oefner P Sachidanandam R Lazebnik Y. Deficiency in glutamine but not glucose induces MYC-dependent apoptosis in human cells. J Cell Biol. (2007) 17:93–105. 10.1083/jcb.200703099 17606868 PMC2064426

[136] Qiao S Koh SB Vivekanandan V Salunke D Patra KC Zaganjor E. REDD1 loss reprograms lipid metabolism to drive progression of RAS mutant tumors. Genes Dev. (2020) 34:751–66. 10.1101/gad.335166.119 32273287 PMC7263146

[137] Biancur DE Paulo JA Małachowska B Quiles Del Rey M Sousa CM Wang X. Compensatory metabolic networks in pancreatic cancers upon perturbation of glutamine metabolism. Nat Commun. (2017) 8:15965. 10.1038/ncomms15965 28671190 PMC5500878

[138] Elgogary A Xu Q Poore B Alt J Zimmermann SC Zhao L. Combination therapy with BPTES nanoparticles and metformin targets the metabolic heterogeneity of pancreatic cancer. Proc Natl Acad Sci USA. (2016) 113:E5328–36. 10.1073/pnas.1611406113 27559084 PMC5018752

[139] Chakrabarti G Moore ZR Luo X Ilcheva M Ali A Padanad M. Targeting glutamine metabolism sensitizes pancreatic cancer to PARP-driven metabolic catastrophe induced by ß-lapachone. Cancer Metab. (2015) 3:12. 10.1186/s40170-015-0137-1 26462257 PMC4601138

[140] Ali U Naveed M Ullah A Ali K Shah SA Fahad S. L-asparaginase as a critical component to combat Acute Lymphoblastic Leukaemia (ALL): a novel approach to target ALL. Eur J Pharmacol. (2016) 771:199–210. 10.1016/j.ejphar.2015.12.023 26698391

[141] Dufour E Gay F Aguera K Scoazec J-Y Horand F Lorenzi PL. Pancreatic tumor sensitivity to plasma L-asparagine starvation. Pancreas. (2012) 41:940–8. 10.1097/MPA.0b013e318247d903 22513289

[142] Nakamura A Nambu T Ebara S Hasegawa Y Toyoshima K Tsuchiya Y. Inhibition of GCN2 sensitizes ASNS-low cancer cells to asparaginase by disrupting the amino acid response. Proc Natl Acad Sci USA. (2018) 115:E7776–85. 10.1073/pnas.1805523115 30061420 PMC6099884

[143] Pathria G Lee JS Hasnis E Tandoc K Scott DA Verma S. Translational reprogramming marks adaptation to asparagine restriction in cancer. Nat Cell Biol. (2019) 21:1590–603. 10.1038/s41556-019-0415-1 31740775 PMC7307327

[144] Bachet J-B Gay F Maréchal R Galais M-P Adenis A MsC DS. Asparagine synthetase expression and phase i study with L-asparaginase encapsulated in red blood cells in patients with pancreatic adenocarcinoma. Pancreas. (2015) 44:1141–7. 10.1097/MPA.0000000000000394 26355551

[145] Hammel P Fabienne P Mineur L Metges J-P Andre T De La Fouchardiere C. Erythrocyte-encapsulated asparaginase (eryaspase) combined with chemotherapy in second-line treatment of advanced pancreatic cancer: an open-label, randomized phase IIb trial. Eur J Cancer. (2020) 124:91–101. 10.1016/j.ejca.2019.10.020 31760314

[146] Bowles TL Kim R Galante J Parsons CM Virudachalam S Kung H-J. Pancreatic cancer cell lines deficient in argininosuccinate synthetase are sensitive to arginine deprivation by arginine deiminase. Int J Cancer. (2008) 123:1950–5. 10.1002/ijc.23723 18661517 PMC4294549

[147] Kim SS Xu S Cui J Poddar S Le TM Hayrapetyan H. Histone deacetylase inhibition is synthetically lethal with arginine deprivation in pancreatic cancers with low argininosuccinate synthetase 1 expression. Theranostics. (2020) 10:829–40. 10.7150/thno.40195 31903153 PMC6929997

[148] Daylami R Muilenburg DJ Virudachalam S Bold RJ. Pegylated arginine deiminase synergistically increases the cytotoxicity of gemcitabine in human pancreatic cancer. J Exp Clin Cancer Res. (2014) 33:102. 10.1186/s13046-014-0102-9 25499121 PMC4279680

[149] Prudner BC Rathore R Robinson AM Godec A Chang SF Hawkins WG. Arginine starvation and docetaxel induce c-Myc-driven hENT1 surface expression to overcome gemcitabine resistance in ASS1-negative tumors. Clin Cancer Res. (2019) 25:5122–34. 10.1158/1078-0432.CCR-19-0206 31113844 PMC7357353

[150] Lowery MA Yu KH Kelsen DP Harding JJ Bomalaski JS Glassman DC. A phase 1/1B trial of ADI-PEG 20 plus nab-paclitaxel and gemcitabine in patients with advanced pancreatic adenocarcinoma. Cancer. (2017) 123:4556–65. 10.1002/cncr.30897 28832976

[151] Singh PK Deorukhkar AA Venkatesulu BP Li X Tailor R Bomalaski JS. exploiting arginine auxotrophy with pegylated arginine deiminase (ADI-PEG20) to sensitize pancreatic cancer to radiotherapy via metabolic dysregulation. Mol Cancer Therap. (2019) 18:2381–93. 10.1158/1535-7163.MCT-18-0708 31395686 PMC6891156

[152] Zheng L Xue J Jaffee EM Habtezion A. Role of immune cells and immune-based therapies in pancreatitis and pancreatic ductal adenocarcinoma. Gastroenterology. (2013) 144:1230–40. 10.1053/j.gastro.2012.12.042 23622132 PMC3641650

[153] Gabrilovich DI Nagaraj S. Myeloid-derived suppressor cells as regulators of the immune system. Nat Rev Immunol. (2009) 9:162–74. 10.1038/nri2506 19197294 PMC2828349

[154] Khaled YS Ammori BJ Elkord E. Increased levels of granulocytic myeloid-derived suppressor cells in peripheral blood and tumour tissue of pancreatic cancer patients. J Immunol Res. (2014) 2014:879897. 10.1155/2014/879897 24741628 PMC3987936

[155] Zhang J Xu X Shi M Chen Y Yu D Zhao C. CD13 neutrophil-like myeloid-derived suppressor cells exert immune suppression through arginase 1 expression in pancreatic ductal adenocarcinoma. Oncoimmunology. (2017) 6:e1258504. 10.1080/2162402X.2016.1258504 28344866 PMC5353902

[156] Prendergast GC Smith C Thomas S Mandik-Nayak L Laury-Kleintop L Metz R. Indoleamine 2,3-dioxygenase pathways of pathogenic inflammation and immune escape in cancer. Cancer Immunol Immunother. (2014) 63:721–35. 10.1007/s00262-014-1549-4 24711084 PMC4384696

[157] Théate I van Baren N Pilotte L Moulin P Larrieu P Renauld J-C. Extensive profiling of the expression of the indoleamine 2,3-dioxygenase 1 protein in normal and tumoral human tissues. Cancer Immunol Res. (2015) 3:161–72. 10.1158/2326-6066.CIR-14-0137 25271151

[158] Zhang T Tan X-L Xu Y Wang Z-Z Xiao C-H Liu R. Expression and prognostic value of indoleamine 2,3-dioxygenase in pancreatic cancer. Chin Med J. (2017) 130:710–6. 10.4103/0366-6999.201613 28303855 PMC5358422

[159] Manuel ER Chen J D'Apuzzo M Lampa MG Kaltcheva TI Thompson CB. Salmonella-based therapy targeting indoleamine 2,3-dioxygenase coupled with enzymatic depletion of tumor hyaluronan induces complete regression of aggressive pancreatic tumors. Cancer Immunol Res. (2015) 3:1096–107. 10.1158/2326-6066.CIR-14-0214 26134178 PMC4561205

[160] Prendergast GC Malachowski WP DuHadaway JB Muller AJ. Discovery of IDO1 inhibitors: from bench to bedside. Cancer Res. (2017) 77:6795–811. 10.1158/0008-5472.CAN-17-2285 29247038 PMC6021761

[161] Pan M Reid MA Lowman XH Kulkarni RP Tran TQ Liu X. Regional glutamine deficiency in tumours promotes dedifferentiation through inhibition of histone demethylation. Nat Cell Biol. (2016) 18:1090–101. 10.1038/ncb3410 27617932 PMC5536113

[162] Maddocks ODK Labuschagne CF Adams PD Vousden KH. Serine metabolism supports the methionine cycle and DNA/RNA methylation through *de novo* ATP synthesis in cancer cells. Mol Cell. (2016) 61:210–21. 10.1016/j.molcel.2015.12.014 26774282 PMC4728077

[163] Ding J Li T Wang X Zhao E Choi J-H Yang L. The histone H3 methyltransferase G9A epigenetically activates the serine-glycine synthesis pathway to sustain cancer cell survival and proliferation. Cell Metab. (2013) 18:896–907. 10.1016/j.cmet.2013.11.004 24315373 PMC3878056

[164] Dann SG Ryskin M Barsotti AM Golas J Shi C Miranda M. Reciprocal regulation of amino acid import and epigenetic state through Lat1 and EZH2. EMBO J. (2015) 34:1773–85. 10.15252/embj.201488166 25979827 PMC4516430

[165] Biancur DE Kimmelman AC. The plasticity of pancreatic cancer metabolism in tumor progression and therapeutic resistance. Biochim Biophys Acta Rev Cancer. (2018) 1870:67–75. 10.1016/j.bbcan.2018.04.011 29702208 PMC6345383

